# Hemorrhage, Disseminated Intravascular Coagulopathy, and Thrombosis Complications Among Critically Ill Patients with COVID-19: An International COVID-19 Critical Care Consortium Study*

**DOI:** 10.1097/CCM.0000000000005798

**Published:** 2023-02-28

**Authors:** Jonathon P. Fanning, Natasha Weaver, Robert B. Fanning, Matthew J. Griffee, Sung-Min Cho, Mauro Panigada, Nchafatso G. Obonyo, Akram M. Zaaqoq, Hannah Rando, Yew Woon Chia, Bingwen Eugene Fan, Declan Sela, Davide Chiumello, Silvia Coppola, Ahmed Labib, Glenn J. R. Whitman, Rakesh C. Arora, Bo S. Kim, Anna Motos, Antoni Torres, Ferran Barbé, Giacomo Grasselli, Alberto Zanella, Eric Etchill, Asad Ali Usman, Maximilian Feth, Nicole M. White, Jacky Y. Suen, Gianluigi Li Bassi, Giles J. Peek, John F. Fraser, Heidi Dalton

**Affiliations:** 1 Critical Care Research Group, The Prince Charles Hospital, Brisbane, QLD, Australia.; 2 Division of Cardiac Surgery, Department of Surgery, Johns Hopkins School of Medicine, Baltimore, MD.; 3 Faculty of Medicine, University of Queensland, Brisbane, QLD, Australia.; 4 Nuffield Department of Population Health, University of Oxford, Oxford, United Kingdom.; 5 School of Medicine and Public Health, The University of Newcastle, Newcastle, NSW, Australia.; 6 Northern Hospital, Northern Health, Melbourne, VIC, Australia.; 7 Faculty of Medicine, University of Melbourne, Melbourne, VIC, Australia.; 8 Department of Anesthesiology and Perioperative Medicine, Sections of Critical Care and Perioperative Echocardiography, University of Utah, Salt Lake City, UT.; 9 Department of Anesthesiology, Anesthesiology Service, Veteran Affairs Medical Center, Salt Lake City, UT.; 10 Division of Neuroscience Critical Care, Department of Neurology and Neurosurgery, Johns Hopkins School of Medicine, Baltimore, MD.; 11 Fondazione IRCCS Ca’ Granda, Ospedale Maggiore Policlinico di Milano, Department of Anesthesia, Intensive Care and Emergency. Milano, Lombardia, Italy.; 12 Initiative to Develop African Research Leaders (IDeAL)/KEMRI-Wellcome Trust Research Programme, Kilifi, Kenya.; 13 Wellcome Trust Centre for Global Health Research, Imperial College London, London, United Kingdom.; 14 Department of Critical Care Medicine, MedStar Washington Hospital Center, Washington, DC.; 15 Department of Medicine, Georgetown University, Washington, DC.; 16 Department of Cardiology, Tan Tock Seng Hospital, Singapore.; 17 Lee Kong Chian School of Medicine, Nanyang Technological University, Singapore.; 18 Yong Loo Lin School of Medicine, National University of Singapore, Singapore.; 19 Department of Haematology, Tan Tock Seng Hospital, Singapore.; 20 Department of Laboratory Medicine, Khoo Teck Puat Hospital, Singapore.; 21 Department of Anesthesia and Intensive Care, Aziende Socio Sanitarie Territoriali (ASST) Santi Paolo e Carlo, San Paolo University Hospital of Milan, Milan, Italy.; 22 Medical Intensive Care Unit, Department of Medicine, Hamad General Hospital, Hamad Medical Corporation, Doha, Qatar.; 23 Section of Cardiac Surgery, Department of Surgery, Max Rady College of Medicine, University of Manitoba, Winnipeg, MB, Canada.; 24 Harrington Heart and Vascular Institute, University Hospitals - Cleveland Medical Center, Cleveland, OH.; 25 Division of Cardiac Surgery, Department of Surgery, Case Western Reserve University, Cleveland, OH.; 26 Centro de Investigación Biomedica En Red – Enfermedades Respiratorias (CIBERES), Madrid, Spain.; 27 Institut d’Investigacions Biomediques August Pi I Sunyer (IDIBAPS), Barcelona, Universitat de Barcelona, Barcelona, Spain.; 28 Servei de Pneumologia, Hospital Clinic, University of Barcelona, Spain.; 29 Institució Catalana de Recerca i Estudis Avançats, Spain.; 30 Translational Research in Respiratory Medicine, Respiratory Department, Hospital Universitari Aranu de Vilanova and Santa Maria, IRBLleida, Leida, Spain.; 31 Department of Pathophysiology and Transplantation, University of Milan, Milan, Italy.; 32 Department of Anesthesia and Critical Care, Hospital of the University of Pennsylvania, University of Pennsylvania, Philadelphia, PA.; 33 Department of Anesthesiology, Critical Care, Emergency and Pain Medicine, Military Medical Center Ulm, Ulm, Germany.; 34 Queensland University of Technology, Brisbane, QLD, Australia.; 35 Congenital Heart Centre, University of Florida, Gainesville, FL.

**Keywords:** COVID-19, extracorporeal membrane oxygenation, hemorrhage, intensive care unit, thrombosis

## Abstract

To determine the prevalence and outcomes associated with hemorrhage, disseminated intravascular coagulopathy, and thrombosis (HECTOR) complications in ICU patients with COVID-19.

**DESIGN:**

Prospective, observational study.

**SETTING:**

Two hundred twenty-nine ICUs across 32 countries.

**PATIENTS:**

Adult patients (≥ 16 yr) admitted to participating ICUs for severe COVID-19 from January 1, 2020, to December 31, 2021.

**INTERVENTIONS:**

None.

**MEASUREMENTS AND MAIN RESULTS:**

HECTOR complications occurred in 1,732 of 11,969 study eligible patients (14%). Acute thrombosis occurred in 1,249 patients (10%), including 712 (57%) with pulmonary embolism, 413 (33%) with myocardial ischemia, 93 (7.4%) with deep vein thrombosis, and 49 (3.9%) with ischemic strokes. Hemorrhagic complications were reported in 579 patients (4.8%), including 276 (48%) with gastrointestinal hemorrhage, 83 (14%) with hemorrhagic stroke, 77 (13%) with pulmonary hemorrhage, and 68 (12%) with hemorrhage associated with extracorporeal membrane oxygenation (ECMO) cannula site. Disseminated intravascular coagulation occurred in 11 patients (0.09%). Univariate analysis showed that diabetes, cardiac and kidney diseases, and ECMO use were risk factors for HECTOR. Among survivors, ICU stay was longer (median days 19 vs 12; *p* < 0.001) for patients with versus without HECTOR, but the hazard of ICU mortality was similar (hazard ratio [HR] 1.01; 95% CI 0.92–1.12; *p* = 0.784) overall, although this hazard was identified when non-ECMO patients were considered (HR 1.13; 95% CI 1.02–1.25; *p* = 0.015). Hemorrhagic complications were associated with an increased hazard of ICU mortality compared to patients without HECTOR complications (HR 1.26; 95% CI 1.09–1.45; *p* = 0.002), whereas thrombosis complications were associated with reduced hazard (HR 0.88; 95% CI 0.79–0.99, *p* = 0.03).

**CONCLUSIONS:**

HECTOR events are frequent complications of severe COVID-19 in ICU patients. Patients receiving ECMO are at particular risk of hemorrhagic complications. Hemorrhagic, but not thrombotic complications, are associated with increased ICU mortality.

**KEY POINTS**

**Question:** What are the prevalences and outcomes associated with hemorrhage, disseminated intravascular coagulopathy, and thrombosis (HECTOR) complications in ICU patients critically ill with COVID-19?

**Findings:** In this large multicenter prospective observational study, a HECTOR complication occurred in 1,732 of 11,969 study eligible patients (14%), with the extracorporeal membrane oxygenation subgroup at particularly high risk. The occurrence of a HECTOR complication was associated with increased ICU mortality, although hazard for mortality was only seen with hemorrhagic complications.

**Meaning:** HECTOR events are frequent complications of severe COVID-19 in ICU patients.

Derangements of the hematologic systems are recognized as key elements in the pathogenesis of severe COVID-19 (1). Early in the pandemic, there were reports of higher-than-expected rates of hemorrhage, disseminated intravascular coagulopathy, and thrombosis (HECTOR), with associated high morbidity and mortality (1–6). However, current data: 1) are limited to small datasets collected from a few centers, 2) have focused primarily on thrombotic complications, and 3) provide little insight into risk factor identification.

Thus, there is an ongoing need for high-quality, large-scale, generalizable, and robust ICU-specific data that focus on COVID-19–associated coagulation/hemostasis changes and resultant thrombosis and hemorrhage. This study was conducted to identify and characterize HECTOR complications among COVID-19 patients requiring ICU admission, including their risk factors, prevalence, and outcomes, to increase our understanding of these complications and develop potential strategies for early diagnosis and risk reduction.

## STUDY DESIGN AND METHODS

### Study Design and Setting

The COVID-19 Critical Care Consortium (CCCC) registry is an ongoing, global database enrolling COVID-19 patients requiring ICU care. Study methods, design, and the rationale behind the CCCC have been published previously (Trial registration ACTRN12620000421932) (7). Hospitals participating in the CCCC obtained approval from their local institutional review board and received waivers of informed consent for all patients. A complete summary of recruiting sites, corresponding ethics/regulatory approvals, contributors, and collaborators is included in **e-Appendices 1–3** (http://links.lww.com/CCM/H289). The study is reported using Strengthening the Reporting of Observational Studies in Epidemiology (STROBE) guidelines (8).

### Participants

The CCCC database was examined for patients admitted to participating ICUs (229 sites across 32 countries) over 2 years from January 1, 2020, to December 31, 2021. Inclusion criteria were as follows: 1) age greater than or equal to 16 years, 2) symptomatic COVID-19 infection (determined by an attending physician) with laboratory confirmation (using real-time polymerase chain reaction/next-generation sequencing), and 3) admission to an ICU for treatment of acute COVID-19. Patients admitted to the ICU for reasons considered unrelated to acute infection with severe acute respiratory syndrome coronavirus 2 (SARS-CoV-2) were excluded.

### Data Collection and Variables

For each patient, data collection began at the time of their admission to the ICU, using the International Severe Acute Respiratory IncideNce sTudy of Severe Acute and Emerging Infection Consortium Short-Period Incidence Study for Severe Acute Respiratory Infection case report forms (CRFs) (9). Further ICU-specific information was gathered using CCCC/extracorporeal membrane oxygenation for 2019 novel coronavirus acute respiratory disease (ECMOCARD) CRFs (10). Deidentified data from each site were uploaded to the Research Electronic Data Capture database, based at University of Oxford, United Kingdom (11). The principle investigator at each site was responsible for ensuring the integrity of the data and submission of the CRFs (**e-Appendix 4**, http://links.lww.com/CCM/H289). Sites were supported by the CCCC coordination center who undertook quality assurance within the data management system and entered case report forms data, monitored for and chased missing data, and provided regular written and web-based training.

For all enrolled patients, the following information was extracted from the CCCC database: demographics, morphometrics, comorbidities, medications, laboratory values, adverse events/complications, and outcomes. Additional case report forms were completed for patients who received mechanical ventilation or extracorporeal membrane oxygenation (ECMO). Disease severity was rated with Acute Physiology and Chronic Health Evaluation (APACHE) II (12) and Sequential Organ Failure Assessment (SOFA) scores at ICU admission (13).

### Definitions

The case report forms utilized for data collection classified HECTOR complications as: 1) thrombosis (ischemic stroke, myocardial ischemia, deep vein thrombosis [DVT], or pulmonary embolism [PE]); 2) hemorrhage which was further classified based on the bleeding source, and when multiple bleeding sources were identified, the two most predominant sources recorded by the site investigators; and 3) disseminated intravascular coagulation (DIC), all as diagnosed by treating clinicians (10). Full definitions used in the case report forms are provided in **e-Appendix 5** (http://links.lww.com/CCM/H289), and a version of the case report form completion guide is provided in e-Appendix 4 (http://links.lww.com/CCM/H289). Patients who suffered HECTOR complications from more than one complication category (i.e., hemorrhage and/or thrombosis and/or DIC) were included in each.

### Study Outcomes

Primary study outcomes were the prevalence of HECTOR complications and each type of thrombosis and hemorrhagic event. Secondary outcomes were ICU mortality at 28 and 90 days and overall, and the duration of their ICU stay, in days.

### Statistical Analysis

The “HECTOR” group was identified as the subset of eligible patients with one or more HECTOR complications recorded in the CCCC database as of March 31, 2022. This group was further classified as subgroups of patients with thrombotic or hemorrhagic complication(s).

Patients with HECTOR complications were compared with patients with no clinical diagnosis of HECTOR (non-HECTOR) in terms of demographic characteristics, past medical history, treatments, duration of treatments, length of stay in ICU/hospital, and outcomes/discharge disposition. Differences between groups (e.g., patients with versus without HECTOR complications) were tested using chi-square test for categorical variables and the Wilcoxon-Mann-Whitney *U* test for continuous variables.

Survival analysis was used to estimate the effect of HECTOR complications (combined, and for thrombotic and hemorrhagic complications separately) on time between ICU admission and mortality in the ICU. The analysis cohort was limited to patients with a nonmissing discharge status and a valid ICU discharge date. Hazard ratios (HRs) for mortality risk were estimated using Cox regression, assuming patients “discharged alive” (alive, home, palliative, hospitalized, or transferred to another facility) were censored independently. The proportional hazards assumption was verified with log-log plots and a test of Schoenfeld residuals. Parametric Weibull regression was also performed as a sensitivity analysis. The Fine-Gray method was used as a second sensitivity analysis to estimate sub-HRs for the cumulative incidence of the competing events of ICU mortality and discharge alive. Each survival analysis method was used to produce crude estimates and adjusted a priori for patient sex, age, body mass index (BMI), any ECMO treatment, and the country where hospitalized. Due to a large proportion of not entered BMI data, all analyses were repeated without adjusting for BMI. Regression results are presented as HRs with 95% CIs and *p* values. The same analyses were prespecified for patients with ECMO data to assess if the requirement for ECMO resulted in over- or underestimation of the impact of HECTOR complications on ICU mortality.

Analysis was performed in SAS 9.4 (SAS Institute Inc., Cary, NC), apart from survival analyses performed in Stata 15 (StataCorp, College Station, TX).

## RESULTS

### HECTOR Prevalence

A total of 17,881 patients were admitted to participating ICUs for COVID-19 management during the study period of whom 11,969 were included in the primary analysis (**e-Fig. 1**, http://links.lww.com/CCM/H289). At least one HECTOR complication occurred in 1,732 patients (14% of the study population). **Table 1** lists the prevalence of each complication. Thrombotic complications occurred in 1,249 patients (10%), with PE (*n* = 712, 57%) and myocardial ischemia (*n* = 413, 33%), the two most frequently observed. Hemorrhagic complications occurred in 579 patients (4.8%), almost half of which involved the gastrointestinal system (*n* = 276, 48%), among which *n* equals to 179 patients (66%) had received steroid treatment. DIC occurred in just 11 patients (< 0.09%). These prevalence values may underestimate true prevalence as it was not possible to differentiate between “no” responses versus missing data for many of the complication variables.

**TABLE 1.** Prevalence of Hemorrhage, Disseminated Intravascular Coagulopathy, and Thrombosis Complications

Complication	Total Cohort (*N* = 11,969)	No-ECMO Requirement (*N* = 10,405)	ECMO Requirement (*N* = 1,162)
All hemorrhage, disseminated intravascular coagulopathy, and thrombosis complications, *n* (%)	1,732 (14)	1,297 (12)	427 (37)
Thrombotic, *n* (%)	1,249 (10% of total sample)	1,057 (10% of no-ECMO)	184 (16% of ECMO)
Pulmonary embolism	712 (57)	635 (60)	72 (39)
Deep vein thrombosis	93 (7.4)	36 (3.4)	57 (31)
Myocardial infarction/cardiac ischemia	413 (33)	373 (35)	37 (20)
Ischemic stroke or cerebrovascular accident	49 (3.9)	44 (4.2)	5 (2.7)
Other thromboembolism	55 (4.4)	19 (1.8)	36 (20)
Hemorrhagic	579 (4.8% of total sample)	295 (2.8% of no-ECMO)	284 (24% of ECMO)
Hemorrhagic complications, site(s), *n* (%)			
Lungs	77 (13)	24 (8.1)	53 (19)
Gastrointestinal	276(48)	179 (61)	97 (34)
Genitourinary	44 (7.6)	24 (8.1)	20 (7.0)
Skin and soft tissue	74 (13)	26 (8.8)	48 (17)
CNS/hemorrhagic stroke	83 (14)	30 (10.2)	53 (19)
Cardiac	5 (0.9)	1 (0.3)	4 (1.4)
ECMO cannula site	68 (12)	0	68 (24)
Iliopsoas	7 (1.2)	0	7 (2.5)
Unknown site	72 (12)	38 (12.9)	34 (12)
Other, *n* (%)	11 (< 0.1% of total sample)	6 (0.1% of no-ECMO)	5 (0.4% of ECMO)
Disseminated intravascular coagulation	11 (100)	6 (100)	5 (100)

ECMO = extracorporeal membrane oxygenation.

Some patients had more than one hemorrhage, disseminated intravascular coagulopathy, and thrombosis complication, so the summed totals of complications exceed the overall number of patients with complications.

### Demographic and Clinical Variables

Demographic and baseline clinical variables for both the HECTOR and non-HECTOR groups are summarized in **Table 2**. The cohort was 69% male, with similar sex distributions in the two groups. Median age (60.0 interquartile range [IQR] 51–68 vs 61.0 yr; IQR 51–70 yr; *p* = 0.03) and BMI (27.8 vs 28.1 kg/m^2^; *p* = 0.03) were not meaningfully different between HECTOR and non-HECTOR patients. HECTOR occurred in 911 of 6,568 of those over 60 (13.9%) versus in 821 of 5,401 among younger (60 yr or less) patients (15.2%) (*p* = 0.039). The younger (median age 51 yr; IQR 42–56 yr) patient subgroup more commonly experienced hemorrhage than the older (median age 69; IQR 65–74) subgroup (6.2% vs 3.8%, respectively; *p* = 0.041). Baseline chronic cardiac disease, chronic kidney disease, diabetes mellitus, and history of cigarette smoking were all more common in HECTOR patients (all *p* < 0.001). Disease severity was higher at ICU admission in patients with HECTOR. Median SOFA scores were 6.0 versus 4.0 (*p* < 0.0001). Median APACHE II scores were 17.0 versus 14.0 (*p* < 0.0001). Clinical management variables are summarized in **Table 3**. Mechanical ventilation was received more frequently in patients with HECTOR (82% vs 73%; *p* < 0.0001) and for a longer duration (median 18.0, IQR 9.0–33.0 vs 13 d, IQR 7.0–25.0 d; *p* < 0.0001). Median ICU stay was also longer in patients with versus without HECTOR (median 18, IQR 8–34 vs 12 d, IQR 6–24 d; *p* < 0.0001). HECTOR patients also more commonly received blood product transfusions (54% vs 43%; *p* < 0.0001), particularly of red cells, fresh frozen plasma, and cryoprecipitate (all *p* < 0.001).

**TABLE 2.** Baseline Patient Characteristics With Accompanying Univariate Analysis

Characteristics	Class or Statistic	HECTOR (*N* = 1,732)	Non-HECTOR (*N* = 1,0237)	*p*
Age (yr)	Median (Q1–Q3)	60.0 (51.0–68.0)	61.0 (51.0–70.0)	0.0292
Body mass index (kg/m2)	Median (Q1–Q3)	27.8 (24.6–32.1)	28.1 (25.1–32.3)	0.0266
Sex, *n* (%)	Male	1,220 (71)	7,032 (69)	0.1414
Ethnicity, *n* (%)	White	356 (34)	1,224 (31)	0.4193
	Black	102 (9.7)	356 (9.1)	
	Asian	285 (27)	1124 (29)	
	Hispanic, aboriginal	153 (15)	621 (16)	
	Other	149 (14)	575 (15)	
Chronic cardiac disease, *n* (%)	Yes	332 (19)	1363 (14)	< 0.0001
Chronic kidney disease, *n* (%)	Yes	176 (10)	753 (7.5)	< 0.0001
Chronic neurologic disorder, *n* (%)	Yes	93 (5.5)	445 (5.2)	0.6619
Chronic hematologic disorder, *n* (%)	Yes	72 (4.2)	345 (4.0)	0.7200
Diabetes, *n* (%)	Yes	380 (25)	1,621 (18)	< 0.0001
Hypertension, *n* (%)	Yes	884 (52)	5,696 (57)	< 0.0001
Smoking, *n* (%)	Never smoked	779 (46)	4,514 (52)	< 0.0001
	Current smoker	451 (27)	2,353 (27)	
	Former smoker	466 (27)	1,783 (21)	
Malignant neoplasm, *n* (%)	Yes	59 (3.5)	314 (3.7)	0.6674
Sequential Organ Failure Assessment score	Median (Q1–Q3)	6.0 (4.0–9.0)	4.0 (3.0–7.0)	< 0.0001
Acute Physiology and Chronic Health Evaluation II score	Median (Q1–Q3)	17.0 (11.0–23.0)	14.0 (9.0–20.0)	< 0.0001

HECTOR = hemorrhage, disseminated intravascular coagulopathy, and thrombosis.

**TABLE 3.** Clinical Management Variables With Accompanying Univariate Analysis

Characteristic	Class or Statistic	HECTOR (*N* = 1,732)	Non-HECTOR (*N* = 1,0237)	*p*
Any invasive ventilation, *n* (%)	Yes	1,422 (82)	7,097 (72)	< 0.0001
Mechanical ventilation, *n* (%)	Yes	1,397 (82)	6,704 (73)	< 0.0001
Mechanical ventilation (d)	Median (Q1–Q3)	18.0 (9.0–33.0)	13.0 (7.0–25.0)	< 0.0001
Time from admission to mechanical ventilation (d)	Median (Q1–Q3)	1.0 (0.0–5.0)	2.0 (0.0–5.0)	0.2015
Prone positioning (mechanical ventilation)	Yes	569 (51)	2,181 (44)	< 0.0001
Prone positioning (before ECMO)	Yes	236 (69)	269 (71)	0. 7273
Inhaled nitric oxide	Yes	154 (14)	259 (5.0)	< 0.0001
Neuromuscular blockade (before ECMO)	Yes	260 (77)	848 (91)	< 0.0001
Tracheostomy	Yes	591 (35)	2141 (24)	< 0.0001
ECMO	Yes	427 (25)	735 (7.5)	< 0.0001
ECMO (d)	Median (Q1–Q3)	18.0 (9.0–32.5)	17.0 (9.0–32.0)	0.8784
Time from admission to ECMO (d)	Median (Q1–Q3)	1.0 (0.0–5.0)	1.0 (0.0–7.0)	0.6207
Vasopressor use	Yes	1,308 (77)	5,087 (62)	< 0.0001
Anticoagulation therapy	Yes	449 (90)	1,604 (91)	0.7299
Transfusion—any blood product	Yes	936 (54)	4,409 (43)	< 0.0001
Transfusion—RBCs	Yes	377 (22)	439 (4.3)	< 0.0001
Transfusion—platelets	Yes	630 (36)	3962 (39)	0.0653
Transfusion—fresh frozen plasma	Yes	97 (5.6)	110 (1.1)	< 0.0001
Transfusion—cryoprecipitates	Yes	36 (2.1)	42 (0.4)	< 0.0001
ICU length of stay (d)	Median (Q1–Q3)	18.0 (8.0–34.0)	12.0 (6.0–24.0)	< 0.0001
Hospital length of stay (d)	Median (Q1–Q3)	26.0 (13.0–46.0)	21.0 (12.0–37.0)	< 0.0001

ECMO = extracorporeal membrane oxygenation, HECTOR = hemorrhage, disseminated intravascular coagulopathy, and thrombosis.

### Mortality and Cause of Death

**Figure 1.**
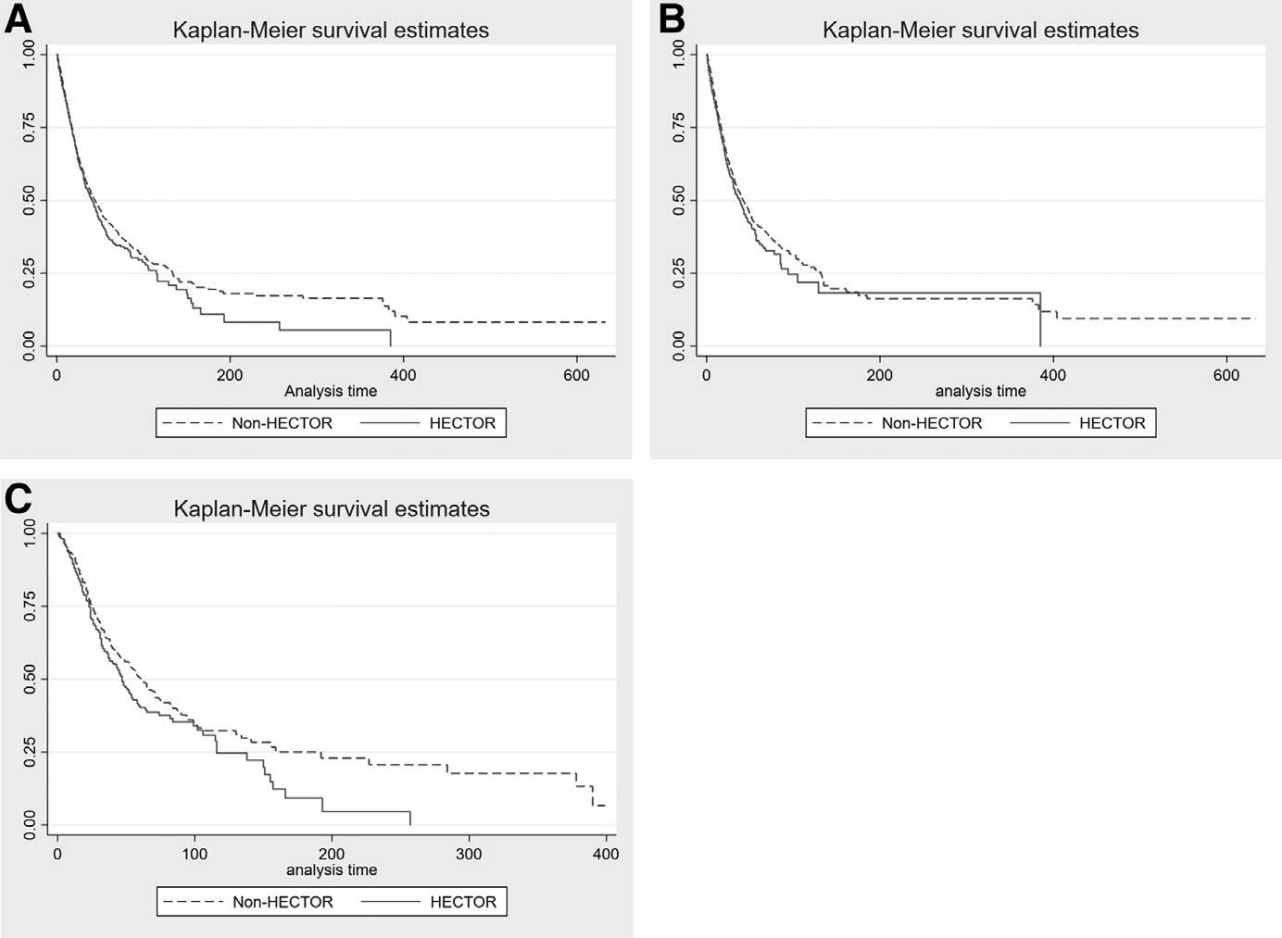
Kaplan-Meier survival curves for hemorrhage, disseminated intravascular coagulopathy, and thrombosis (HECTOR) (*solid line*) versus non-HECTOR (*dashed line*) patients forcohort overall (**A**), patients who were known to not have received extracorporeal membrane oxygenation (ECMO) (**B**), and patients who were known to have received ECMO (**C**).

In our cohort, 4,425 patients (37%) died in the ICU, with a significantly higher rate of death in patients with versus without HECTOR complications (44% vs 36%; *p* < 0.0001) (**Table 4**). Respiratory failure was the most common cause (33%) of death in non-HECTOR patients, whereas multiple organ failure was the most common cause of death (36%) in HECTOR patients (**e-Table 1**, http://links.lww.com/CCM/H289). The Kaplan-Meier survival curves depict not statistically significant reduced survival for patients with versus without HECTOR complications (log-rank *p* = 0.06) (**Fig. 1*A***) and thrombotic (vs no thrombotic) complication subgroup (log-rank *p* = 0.51) (**e-Fig. 2*A***, http://links.lww.com/CCM/H289), but a significant difference between survivor function for hemorrhagic (vs no hemorrhagic) complication subgroup (log-rank test statistic 15.94, *p* = 0.0001) (**e-Fig. 2*B***, http://links.lww.com/CCM/H289). This was confirmed in those known to have not received ECMO support (**Fig. 1*B***).

**TABLE 4.** Patient Disposition Outcomes

Characteristics	Class or Statistic	HECTOR (*N* = 1,732)	Non-HECTOR (*N* = 10,237)	*p*
Mortality at 28 d, *n* (%)	Yes	421 (25)	1,312 (13)	< 0.0001
Mortality at 90 d, *n* (%)	Yes	549 (32)	1,498 (15)	< 0.0001
Discharge disposition, *n* (%)	Discharged dead	755 (44)	3,670 (36)	< 0.0001
	Discharged alive	777 (45)	5,881 (57)	
	Hospitalization	63 (3.6)	259 (2.5)	
	Transferred to other facility	132 (7.6)	407 (4.0)	
	Palliative discharge	5 (0.3)	20 (0.2)	
Time from ICU admission to death (d)	Median (Q1–Q3)	15.0 (6.0–28.0)	11.0 (5.0–21.0)	< 0.0001
Time from ICU admission to discharge alive (d)	Median (Q1–Q3)	19.0 (9.0–38.0)	12.0 (6.0–25.0)	< 0.0001
Time from admission to death (d)	Median (Q1–Q3)	16.0 (7.0–29.0)	13.0 (6.0–23.0)	0.0021
Time from admission to discharge alive (d)	Median (Q1–Q3)	32.0 (18.0–53.0)	22.0 (13.0–39.0)	< 0.0001

ECMO = extracorporeal membrane oxygenation, HECTOR = hemorrhage, disseminated intravascular coagulopathy, and thrombosis.

Mortality in the ICU was 57% for patients with hemorrhagic complications and 53% for patients with thrombotic complications. Among hemorrhagic complications, the highest mortality rate was observed for hemorrhagic stroke (75%) (**e-Tables 2** and **3**, http://links.lww.com/CCM/H289). Among thrombotic complications, the highest mortality rates accompanied ischemic stroke and myocardial ischemia (both 53%) (**e-Table 4**, http://links.lww.com/CCM/H289). Mortality in participants with both hemorrhagic and thrombotic events (*n* = 95) was not significantly increased compared with those in whom only a hemorrhagic complication was reported for ICU (54% vs 58%), 28-day (35% vs 42%), and 90-day (52% vs 57%) mortality. Substantial geographic variation was evident, with country-specific mortality ranging from 13% to 56% (**e-Table 5**, http://links.lww.com/CCM/H289). DIC carried a high mortality, with seven of nine patients (82%) dying in the ICU. HRs for ICU mortality (**Table 5**; and **e-Table 6**, http://links.lww.com/CCM/H289), estimated via Cox regression (adjusted for sex, age, BMI, ECMO, and country), showed that having a HECTOR complication was not associated with an overall increased hazard of death (HR 1.01; 95% CI 0.92–1.12; *p* = 0.784). This lack of any association resulted from the increased hazard of ICU mortality associated with hemorrhagic complications (HR 1.26; 95% CI 1.09–1.45; *p* = 0.002) being sufficiently offset by a reduced hazard of ICU mortality associated with thrombotic complications (HR 0.88; 95% CI 0.79–0.99; *p* = 0.03). Our assessment of the proportional hazards assumption holds (**e-Fig. 3**, http://links.lww.com/CCM/H289). Due to missing data, further adjusting for disease severity was only possible via inclusion of the surrogate measure “vasopressor use” (**e-Table 7**, http://links.lww.com/CCM/H289), which confirmed overall HECTOR was at least not associated with hazard for ICU mortality, although overinterpretation of this analysis should be avoided due to greater than 10% loss of sample size from the regression model. Additional sensitivity analyses stemming from country-specific Cox regression (**e-Table 8**, http://links.lww.com/CCM/H289), cumulative incidence Fine-Gray competing risks and parametric Weibull regression analyses (**e-Tables** 6 and **9**, http://links.lww.com/CCM/H289), and adjustment for BMI (e-Table 9, http://links.lww.com/CCM/H289) generally support the aforementioned findings within the constraints of reduced power in these versus primary model.

**TABLE 5.** Analysis of Time-To-Death/Discharge Using Cause-Specific Hazard Ratios for ICU Mortality Estimated Via Cox Regression

Cohort	Unadjusted Hazard Ratio (95% CI)	Unadjusted *p*	Adjusted^a^ Hazard Ratio (95% CI)	Adjusted^a^ *p*
Overall cohort (*N* = 11,969)				
HECTOR	1.08 (1.00–1.18)	0.062	1.01 (0.92–1.12)	0.784
HECTOR—thrombotic	0.97 (0.87–1.07)	0.514	0.88 (0.79–0.99)	0.030
HECTOR—hemorrhagic	1.28 (1.13–1.44)	< 0.001	1.26 (1.09–1.45)	0.002
No-ECMO required (*n* = 10,400/11,567)^b^				
HECTOR	1.13 (1.02–1.25)	0.015	1.12 (1.01–1.24)	0.029
HECTOR—thrombotic	1.03 (0.92–1.15)	0.613	1.03 (1.03–1.03)	0.869
HECTOR—hemorrhagic	1.53 (1.29–1.82)	< 0.001	1.57 (1.32–1.87)	< 0.001
ECMO required (*n* = 1,162/11,567)				
HECTOR	1.24 (1.04–1.49)	0.018	1.18 (0.99–1.42)	0.070
HECTOR—thrombotic	0.80 (0.62–1.03)	0.082	0.78 (0.61–1.00)	0.049
HECTOR—hemorrhagic	1.50 (1.24–1.81)	< 0.001	1.42 (1.17–1.73)	< 0.001

ECMO = extracorporeal membrane oxygenation, HECTOR = hemorrhage, disseminated intravascular coagulopathy, and thrombosis.

a Adjusted for sex, age, and, ECMO required (for the overall cohort only, not “No ECMO required” or “ECMO required” subgroups)

b Denominator differs from overall cohort due to missing ECMO requirement (*n* = 402). These analyses were adjusted for sex and age.

Patients who were discharged alive are assumed to be censored, model assumes independent censoring and proportional hazards, no assumptions on form of baseline hazard function. The adjusted effects are from shared frailty models (which include a random effect for country; all other covariates are fixed effects).

### Patients Requiring ECMO Support

Approximately 10% of the patients received ECMO (*n* = 1,162/11,567 patients for whom ECMO data were available) (e-Fig. 1, http://links.lww.com/CCM/H289), which was venous-venous in greater than 90%. Chronic cardiovascular disease, diabetes, and White ethnicity/race were the only baseline characteristics that were meaningfully different in patients requiring ECMO who experienced HECTOR complications (**e-Table 10**, http://links.lww.com/CCM/H289). ECMO support during ICU admission was significantly higher in HECTOR than non-HECTOR patients (25% vs 7.5%; *p* < 0.001). Among those patients who received ECMO, 37% (427/1,162) experienced some HECTOR complication, versus only 12% of patients (1,297/10,405) who did not receive ECMO (*p* < 0.001) (Table 1) (e-Table 10, http://links.lww.com/CCM/H289). Most HECTOR complications in ECMO patients were hemorrhagic 67% (*n* = 284/427), compared with only 23% (*n* = 295/1,297) in patients not requiring ECMO (*p* < 0.001). Of patients requiring ECMO, 50% (*n* = 580/1,297) died in the ICU versus 35% of patients (*n* = 3,652/10,405) not requiring ECMO. Among patients who received ECMO, mortality was 21% (*n* = 240/1,162) in those with versus 29% (340/1,162) without HECTOR complications (**e-Table 11**, http://links.lww.com/CCM/H289). The cause of death was similar between ECMO patients who did versus did not have HECTOR complications (**e-Table 12**, http://links.lww.com/CCM/H289). The Kaplan-Meier survival curves show significant difference between those ECMO-requiring patients with HECTOR complications and those without (**Fig. 1*C***). Adjusted HR for ICU mortality (HR 1.18; 95% CI 0.99–1.42; *p* = 0.07) was also nonsignificant; however, this balanced a reduced hazard for ICU mortality with thrombosis (HR 0.78; 95% CI 0.61–1.00; *p* = 0.049) and an increased hazard for hemorrhage (HR 1.42; 95% CI 1.17–1.73; *p* < 0.001) (Table 5).

## DISCUSSION

In this global, multicenter database analysis, hemorrhagic and thrombotic complications were common (14%) in critically ill patients with COVID-19, exceeding reports of similar complications in other respiratory virus infections such as influenza (14). The presence of one or more HECTOR complications was also associated with increased ICU mortality. Key risk factors for HECTOR complications were baseline chronic medical conditions (diabetes, chronic cardiac disease, and chronic kidney disease), a smoking history, and high APACHE II and SOFA scores on admission to the ICU. These findings highlight the importance of identifying HECTOR complications in patients with COVID-19 and emphasize the need for future studies on strategies to mitigate these complications.

Observed prevalence of thrombotic complications was less than previously reported, occurring in only 10% of our cohort. Meta-analyses report the frequency of venous thromboembolism in the ICU between 23% and 31% and arterial thrombotic events in 5% (5, 6, 15). Regarding hemorrhagic complications, one clinical trial assessing anticoagulation in critically ill COVID-19 patients identified major bleeding in 3.8% of patients assigned to therapeutic-dose anticoagulation versus 2.3% of patients receiving usual care thromboprophylaxis compared with 4.9% reported here (16).

There may be several reasons for our results differing from existing literature. Methodological differences that could explain the difference between other studies and our own include how complications were defined and detected (i.e., protocolized routine screening vs clinical diagnosis) and whether these other studies were retrospective or prospective, reducing data comparability across studies (15). Geographic and temporal differences also may exist in resource availability and clinical practices, including the availability of antiviral therapy (e.g., remdesivir, nirmatrelvir, molnupiravir), monoclonal antibodies (e.g., sotrovimab), varying intensities of antithrombotic use, and screening/diagnostic investigations (16–18). Publication bias resulting in previous overestimation of thrombosis may also be a contributing factor (5).

Patients receiving ECMO for COVID-19–related severe ARDS were at particularly high risk of hemorrhagic complications (67%) compared with the previously reported incidence of hemorrhagic events in non–COVID-19 ECMO patients (29.3%) (19). Several factors could explain this, including the high disease severity, anticoagulation, an intense systemic inflammatory response at presentation, and hemostatic changes inherent to ECMO (20). Previous estimates indicating increased circuit clotting, a higher-than-expected occurrence of on-ECMO PE, and hemorrhagic stroke may not persist when these estimates are corrected for ECMO run duration (typically longer in COVID-19 patients) (21–24). Studies have shown that mortality in COVID-19 patients on ECMO is not substantially different than in patients on ECMO for ARDS caused by other respiratory viruses (24–26). However, HECTOR complicating severe COVID-19 requiring ECMO is reported at higher rates here than in many previous reports in patients requiring ECMO for both COVID and non-COVID indications, although not as frequently as in the 2022 report from the French national ECMOSARS registry (*n* = 620) (27).

DIC accounts for a small but important number of COVID-19–associated coagulopathies that predispose patients to both thrombosis and bleeding. We found DIC to be a rare complication that carried a high mortality, occurring in 0.09% of our cohort. Whether this is a true reflection of lack of occurrence or a data collection/underreporting issue cannot be determined. Furthermore, the small size of the DIC group limited survival analysis. This contrasts with an early meta-analysis that revealed the prevalence of DIC to be approximately 3% (28). In another study, nearly 70% of nonsurvivors may have met criteria for DIC highlighting the potential importance of this complication (29).

Most patients in the dataset only experienced one type of HECTOR complication (thrombotic or hemorrhagic), with under 10% of HECTOR patients experiencing both. It is, therefore, conceivable that different HECTOR complications result from distinct hemostatic and biochemical phenotypes in response to the inflammatory state associated with severe COVID-19 infection. Further research and matched analyses of relevant biomarkers may help elucidate each profile’s characteristics and risk factors. The most common site of hemorrhage was gastrointestinal. An important future research question is whether gastrointestinal hemorrhage reflects direct effects of SARS-CoV-2 in the gut, with a distinct disease course from SARS-CoV-2 primarily affecting the lungs, or an interaction of the gut immune response and the overall host response to COVID-19 (30, 31).

The ICU mortality following a HECTOR complication was high compared with patients without HECTOR complications (44% vs 36%; *p* < 0.0001). Although not specific to ICU settings, in one meta-analysis, having a thromboembolic complication increased the pooled odds of mortality by almost 75% in hospitalized COVID-19 patients (6). Certainly, in our analysis, patients with HECTOR complications were sicker upon admission to the ICU, as reflected by their SOFA and APACHE II scores. As expected, HECTOR patients were more likely to be “discharged dead” from the ICU, with 28-day (vs 90-d) ICU mortality mostly contributing to this. Interestingly, only hemorrhagic complications were associated with a higher HR for mortality. Several factors may account for this, including increased blood product requirements and the comparative difficulty managing some hemorrhagic complications, for example, intracranial hemorrhage with associated high mortality, compared with thrombotic events (32). Conversely, the reduced hazard for ICU mortality associated with thrombotic complications may reflect the established increased risk of thrombosis (notably DVT and PE) that prolonged time in an ICU confers or other confounding factors that have not been adjusted for (33). Alternatively, differing COVID-19 phenotypes may predispose patients to HECTOR complications but also have survival benefits (34). Mortality analysis in our study is limited by missing data (to different degrees across different centers) restricting adjustment of the regression models for factors that may confound the relationship between HECTOR complications and mortality. Consequently, our analyses should be considered in a hypothesis-generating light.

This study has several limitations. Key among them is missing data. Although 17,881 patients were recorded in the registry (e-Fig. 1, http://links.lww.com/CCM/H289), only 11,969 could be included in our primary analysis, with the primary reason for exclusion being either missing admission or discharge date. This is most evident for Spain and Italy where final survival outcome is unknown (in 24% and 43%, respectively) despite a recorded discharge date resulting in implausibly low mortality rates. Furthermore, a high percentage of data was missing on disease severity (SOFA and APACHE II scores), anticoagulation variables, certain sites of thrombosis (e.g., central venous catheter or ECMO circuit associated), and select laboratory measures which limited our ability to account for, or report on, these variables (**e-Tables 13** and **14**, http://links.lww.com/CCM/H289). Our results may also be skewed by the overrepresentation of certain countries, such as Spain (*n* = 5,269) (e-Tables 5, 8, and 14, http://links.lww.com/CCM/H289). Data analyzed were extracted from the database retrospectively and by numerous researchers. Standardized case report forms were used to minimize the degree of reporting variability (e-Appendix 5, http://links.lww.com/CCM/H289). However, form completion was prone to heterogeneity from variations in geographical and institutional screening and treatment policies, particularly pertinent for thrombotic and bleeding complications. Similarly, access to resources like ECMO is variable. Some patients in our data set may have had indications for ECMO but have been unable to access it due to differences, for example, in ECMO availability and initiation criteria. The degree to which HECTOR complications are a marker of high disease severity rather than a direct contributor to ICU mortality requires further investigation. Finally, this study reports inpatient data up to the ICU discharge date. Except for mortality, outcomes beyond that are unknown. This may be most relevant to thrombosis complications where prevalence is anticipated to increase with time and, as such, events missed.

## CONCLUSIONS

In an international registry of critically ill COVID-19 patients, HECTOR complications were common, affecting 14% of patients. Our results provide data broadly applicable to many healthcare settings globally. The occurrence of a HECTOR complication was associated with longer lengths of stay in the ICU and longer duration of mechanical ventilation relative to patients with no HECTOR complications. Hemorrhagic, but not thrombotic, complications were associated with increased hazard for mortality in the ICU. Patients receiving ECMO seem at particularly high risk of HECTOR complications, most notably hemorrhagic complications. Risk factors for developing a HECTOR complication include preexisting conditions such as diabetes mellitus, hypertension, chronic cardiac and kidney disease, and cigarette smoking, as well as greater disease severity on admission to the ICU (using APACHE II or SOFA scores).

## Supplementary Material


