# Supplementary material for: Hemorrhage, Disseminated Intravascular Coagulopathy, and Thrombosis Complications Among Critically Ill Patients with COVID-19: An International COVID-19 Critical Care Consortium Study*
Source: Crit Care Med. 2023 Feb 28;51(5):619–31. doi: 10.1097/CCM.0000000000005798 (PMC10089926; doi:10.1097/CCM.0000000000005798)
Supplement: Supplementary file 1 [file ccm-51-0619-s001.docx]

**SUPPLEMENTARY MATERIAL**

**Title:** Hemorrhage, Disseminated Intravascular Coagulopathy, and Thrombosis (HECTOR) Complications Among Critically Ill Patients with COVID-19: An International COVID-19 Critical Care Consortium Study

**Short title/running head:** Thrombotic and hemorrhagic complications of COVID-19

**Authors:**

Jonathon P. **Fanning**, MBBS, PhD, FRACP, FANZCA, FCICM^1,2,3,4^; Natasha **Weaver**, PhD*^1,5^; Robert B. **Fanning**, BBiomed, MD*^6,7^; Matthew J. **Griffee**, MD^8,9^; Sung-Min **Cho**, DO, MHS^2,10^; Mauro **Panigada**, MD^11,12^; Nchafatso G. **Obonyo**, MD, PhD, DTM&H^1,3,13,14^; Akram M. **Zaaqoq** MD, MPH^15,16^; Hannah **Rando,** MD, MPH^2^; Yew Woon **Chia**, MBBS, FRCP(Edin), EDIC, DDU^17,18,19^; Bingwen Eugene **Fan**, MBBS, MRCP, MMed^18,19,20,21^; Declan **Sela** BSc, MBBS^1,3^; Davide **Chiumello**, MD^22^; Silvia **Coppola**, MD^22^; Ahmed **Labib**, MBBCh(Hons), FRCA, FFICM^23^; Glenn JR **Whitman**, MD^2^; Rakesh C. **Arora**, MD, PhD, FACS, FRCSC^24^; Bo S. **Kim,** MD^2^; Anna **Motos**^25,26^; Antoni **Torres** MD, PhD^25,26^; Ferran **Barbé**, MD, PhD^25,27^; Giacomo **Grasselli**, MD^11,12^; Alberto **Zanella**, MD^11,12^; Eric **Etchill**, MD, MPH^2,28^; Asad Ali **Usman**, MMD, MPH^29^; Nicole **White**, PhD^1,30^; Jacky **Suen**, PhD^1,3^; Gianluigi **Li Bassi**, MD, PhD^1,3,30,31^; Giles J. **Peek**, MD, FRCS, FFICM, FELSO^32^; John F. **Fraser**, MBChB, PhD, FRCP(Glas), FRCA, FFARCSI, FCICM, FELSO^1,3,30,31^; Heidi **Dalton**, MD, MCCM, FELSO^33,16^; *on behalf of* *the COVID-19 Critical Care Consortium*.

*contributed equally to the manuscript

**Affiliations:**

1. Critical Care Research Group, The Prince Charles Hospital, Brisbane, Australia
2. Division of Cardiac Surgery, Department of Surgery, Johns Hopkins School of Medicine, Baltimore, Maryland, United States of America
3. Faculty of Medicine, University of Queensland, Brisbane, Australia
4. Nuffield Department of Population Health, University of Oxford, United Kingdom
5. School of Medicine and Public Health, The University of Newcastle, New South Wales, Australia
6. Northern Hospital, Northern Health, Melbourne, Victoria, Australia
7. Faculty of Medicine, University of Melbourne, Victoria, Australia
8. Department of Anesthesiology and Perioperative Medicine, Sections of Critical Care and Perioperative Echocardiography, University of Utah, Salt Lake City, Utah, United States of America
9. Anesthesiology Service, Veteran Affairs Medical Center, Salt Lake City, Utah, United States of America
10. Division of Neuroscience Critical Care, Department of Neurology and Neurosurgery, Johns Hopkins School of Medicine, Baltimore, Maryland, United States of America
11. Fondazione IRCCS Ca’ Granda, Ospedale Maggiore Policlinico di Milano, Department of Anesthesia, Intensive Care and Emergency. Milano, Lombardia, Italy
12. University of Milan, Milan, Italy
13. Initiative to Develop African Research Leaders (IDeAL)/KEMRI-Wellcome Trust Research Programme, Kilifi, Kenya
14. Wellcome Trust Centre for Global Health Research, Imperial College London, United Kingdom
15. Department of Critical Care Medicine, MedStar Washington Hospital Center, Washington, District of Columbia, United States of America
16. Georgetown University, Washington, District of Columbia, United States of America
17. Department of Cardiology, Tan Tock Seng Hospital, Singapore
18. Lee Kong Chian School of Medicine, Nanyang Technological University, Singapore
19. Yong Loo Lin School of Medicine, National University of Singapore, Singapore
20. Department of Haematology, Tan Tock Seng Hospital, Singapore
21. Department of Laboratory Medicine, Khoo Teck Puat Hospital, Singapore
22. Department of Anesthesia and Intensive Care, Aziende Socio Sanitarie Territoriali (ASST) Santi Paolo e Carlo, San Paolo University Hospital of Milan, Milan, Italy.
23. Medical Intensive Care Unit, Department of Medicine, Hamad General Hospital, Hamad Medical Corporation, Doha, Qatar
24. Section of Cardiac Surgery, Department of Surgery, Max Rady College of Medicine, University of Manitoba, Winnipeg, Manitoba, Canada
25. Centro de Investigación Biomedica En Red – Enfermedades Respiratorias (CIBERES), Barcelona, Spain
26. Institut d’Investigacions Biooediques August Pi I Sunyer (IDIBAPS), Barcelona, Universitat de Barcelona, Barcelona, Spain
27. Translational Research in Respiratory Medicine, Respiratory Department, Hospital Universitari Aranu de Vilanova and Santa Maria, IRBLleida, Leida, Spain
28. Division of Cardiothoracic Surgery, Department of Surgery, University of Colorado, Colorado, United States of America
29. Department of Anesthesia and Critical Care, Hospital of the University of Pennsylvania, University of Pennsylvania, Philadelphia, Pennsylvania, United States of America
30. Queensland University of Technology, Queensland, Australia
31. Department of Intensive Care, St Andrew’s War Memorial Hospital, UnitingCare Health, Spring Hill, Queensland, Australia
32. Congenital Heart Centre, University of Florida, Gainesville, Florida, United States of America
33. Heart and Vascular Institute, Inova Fairfax Hospital, Falls Church, Virginia, United States of America

**Corresponding Author:**

Jonathon P. Fanning

Critical Care Research Group

Level 3, Clinical Sciences Building, The Prince Charles Hospital

Chermside, 4032, Queensland, Australia

Email: [j.fanning@uq.edu.au](mailto:j.fanning@uq.edu.au)

**SUPPLEMENTARY TABLES**

**e-Table 1:** Cause of death for patients with in-hospital mortality (by HECTOR)

**e-Table 2:** ICU discharge disposition and mortality outcomes (hemorrhagic HECTOR complications)

**e-Table 3:** Mortality incidence for each of the major categories of complication within the hemorrhagic subgroup of HECTOR complications

**e-Table 4:** ICU discharge disposition and mortality outcomes (thrombotic subgroup of HECTOR complications)

**e-Table 5:** Country-specific mortality for selected countries – 5 most represented countries in the dataset (and Australia)

**e-Table 6:** Sensitivity analyses of time-to-death/discharge using hazard ratios for ICU mortality estimated via Cox regression and via parametric Weibull regression and cumulative incidence sub-hazard ratios estimated via the Fine-Gray method. All models adjusted for: patient age, sex, BMI, any ECMO and country

**e-Table 7:** Sensitivity analyses of time-to-death/discharge using hazard ratios for ICU mortality estimated via Cox regression adjusted for: patient age, sex, any ECMO, country and vasopressor use

**e-Table 8:** Country-specific analysis of time-to-death/discharge for the 5 most represented countries using hazard ratios for ICU mortality estimated via Cox regression adjusted for: patient age, sex, BMI, ECMO (no shared frailty for country)

**e-Table 9:** Sensitivity analyses of time-to-death/discharge using hazard ratios for ICU mortality estimated via Cox regression and via parametric Weibull regression and cumulative incidence sub-hazard ratios estimated via the Fine-Gray method. All models adjusted for: patient age, sex, any ECMO, and country.

**e-Table 10:** Baseline patient characteristics with accompanying univariate analysis for patients that were known have required ECMO and those who did not. SOFA, Sequential Organ Failure Assessment; APACHE, Acute Physiology and Chronic Health Evaluation.

**e-Table 11:** Disposition outcomes for patients requiring ECMO

**e-Table 12:** Cause of death for patients requiring ECMO with ICU mortality (by HECTOR)

**e-Table 13:** Data completeness

**e-Table 14:** Data completeness (stratified by country)

**SUPPLEMENTARY FIGURES**

**e-Figure 1:** STROBE patient flow chart

**e-Figure 2:** Kaplan-Meier survival curves for HECTOR subgroups.

**e-Figure 3:** Log-log plot with HECTOR (red) and non-HECTOR (blue) groups.

**SUPPLEMENTARY APPENDICES**

**e-Appendix 1:** Full list of recruiting sites included in the CCCC and relevant IRB approvals

**e-Appendix 2:** List of contributors

**e-Appendix 3:** List of collaborators

**e-Appendix 4:** Case report form

**e-Appendix 5:** Case report definitions for HECTOR complications

**e-Tables**

**e-Table 1:** Cause of death for patients with in-hospital mortality (by HECTOR)

| **Cause of Death  n (column%)** | **Non-HECTOR group**  **(n=3670)** | **HECTOR group**  **(n=755)** | **Total deaths (n=4425)** |
| --- | --- | --- | --- |
| Respiratory failure | 1220 (33%) | 217 (29%) | 1438 |
| Multi-organ failure | 1052 (29%) | 271 (36%) | 1323 |
| Unknown* | 802 (22%) | 24 (3.4%) | 838 |
| Septic shock | 226 (6.2%) | 74 (9.8%) | 300 |
| “Other” | 179 (4.9%) | 57 (7.5%) | 236 |
| Cardiac failure | 109 (3.0%) | 57 (7.5%) | 166 |
| Hemorrhagic shock | 31 (0.8%) | 11 (1.5%) | 42 |
| Cardiovascular accident | 21 (0.6%) | 11 (1.5%) | 32 |
| Cerebrovascular accident | 16 (0.4%) | 27 (3.6%) | 43 |
| Liver failure | 3 (0.1%) | 3 (0.4%) | 6 |
| Treatment withdrawn, prognosis poor | 1 (0.03%) | 0 (0.0%) | 1 |

*includes n=12 listed as “Not applicable”

**e-Table 2:** ICU discharge disposition and mortality outcomes (hemorrhagic HECTOR complications)

| **Outcome** | **Level** | **Any hemorrhagic complication (n=579)** | **CNS/hemorrhagic stroke (n=83)** | **Received transfusion* (n=5345)** | **No hemorrhagic complication (n=11390)** |
| --- | --- | --- | --- | --- | --- |
| Discharge disposition | Discharged alive | 135 (23%) | 10 (12%) | 3286 (61%) | 6523 (57%) |
|  | Discharged dead | 332 (57%) | 62 (75%) | 1865 (35%) | 4093 (36%) |
|  | Hospitalization | 36 (6.2%) | 1 (1.2%) | 50 (0.9%) | 286 (2.5%) |
|  | Palliative discharge | 2 (0.3%) | 1 (1.2%) | 3 (0.1%) | 23 (0.2%) |
|  | Transferred to other facility | 74 (13%) | 9 (11%) | 141 (2.6%) | 465 (4.1%) |
| Mortality at 28 days | Yes | 232 (41%) | 48 (62%) | 313 (5.9%) | 1501 (13%) |
| Mortality at 90 days | Yes | 317 (56%) | 57 (73%) | 429 (8.0%) | 1730 (15%) |

* Received transfusion of any blood product

**e-Table 3:** Mortality incidence for each of the major categories of complication within the hemorrhagic subgroup of HECTOR complications.

| **Site of hemorrhage** | **ICU mortality** | **28-day mortality** | **90-day mortality** |
| --- | --- | --- | --- |
| Lungs (n=77) | 45 (58%) | 28 (63%) | 44 (58%) |
| Gastrointestional (n=276) | 152 (55%) | 101 (37%) | 142 (53%) |
| Genitourinary (n=44) | 22 (50%) | 16 (36%) | 22 (50%) |
| Skin/soft tissue (n=74) | 39 (53%) | 23 (31%) | 39 (53%) |
| CNS/hem stroke (n=83) | 62 (75%) | 48 (62%) | 57 (73%) |
| Cardiac (n=5) | 2 (40%) | 2 (40%) | 2 (40%) |
| ECMO cannula site (n=68) | 42 (62%) | 23 (34%) | 39 (57%) |
| Iliopsoas (n=7) | 6 (86%) | 4 (57%) | 6 (86%) |
| Unknown (n=72) | 35 (49%) | 25 (35%) | 35 (49%) |

**e-Table 4:** ICU discharge disposition and mortality outcomes (thrombotic subgroup of HECTOR complications)

| **Outcome** | **Level** | **Any thrombotic complication (n=1249)** | **Subtypes of thrombotic complication** | | | | **No thrombotic complication (n=10720)** |
| --- | --- | --- | --- | --- | --- | --- | --- |
|  |  |  | **Pulmonary embolism (n=712)** | **Ischemic stroke (n=49)** | **Myocardial ischemia (n=413)** | **Any OTHER thrombotic complication (n=114)** |  |
| Discharge disposition | Discharged alive | 475 (38%) | 486 (66%) | 12 (24%) | 156 (38%) | 49 (43%) | 5990 (56%) |
|  | Discharged dead | 668 (53%) | 215 (30%) | 26 (53%) | 219 (53%) | 35 (31%) | 3950 (37%) |
|  | Hospitalization | 32 (2.6%) | 7 (0.1%) | 2 (4.1%) | 17 (4.1%) | 6 (5.3%) | 290 (2.7%) |
|  | Palliative discharge | 4 (0.3%) | 0 (0%) | 2 (4.1%) | 1 (0.2%) | 1 (0.9%) | 21 (0.2%) |
|  | Transferred to other facility | 70 (5.6%) | 22 (3.1%) | 7 (14%) | 20 (4.8%) | 23 (20%) | 469 (4.4%) |
| Mortality at 28 days | Yes | 222 (18%) | 67 (9.4%) | 22 (46%) | 123 (31%) | 19 (17%) | 1511 (14%) |
| Mortality at 90 days | Yes | 281 (23%) | 91 (13%) | 25 (52%) | 146 (36%) | 32 (28%) | 1766 (17%) |

**e-Table 5:** Country-specific mortality for selected countries – 5 most represented countries in the dataset (and Australia). The comparison highlights the geographic variation seen within the database. Median (IQR) for numeric variables and n(%) for categorical variables are presented. BMI, body mass index; OR, odds ratio.

|  | **Australia (n=198)** | **India (n=570)** | **Indonesia (n=644)** | **United States (n=1155)** | **Italy (n=1876)** | **Spain (n=5296)** |
| --- | --- | --- | --- | --- | --- | --- |
| Age in years | 55 (38, 66) | 59 (45, 68) | 55 (47, 64) | 59 (49, 71) | 63 (55, 70) | 63 (54, 71) |
| Sex - Male | 125 (63%) | 378 (66%) | 415 (64%) | 693 (60%) | 1449 (77%) | 3715 (70%) |
| BMI | 29.2 (25.5, 35.5) | 23.8 (22.5, 24.2) | 24.8 (22.5, 28.0) | 31.6 (26.9, 37.2) | 27.8 (25.5, 31.3) | 28.7 (26.0, 32.1) |
| In-hospital mortality within 28 days | 19 (9.6%) | 159 (28%) | 342 (53%) | 333 (29%) | 159 (8.5%) | 60 (1.1%) |
| Discharged dead | 26 (13%) | 175 (31%) | 361 (56%) | 444 (38%) | 834 (45%) | 1653 (31%) |
| Time from ICU admission to discharged dead (days) | 17.0 (8.0, 21.0) | 7.5 (3.5, 12.0) | 5.0 (2.0, 8.5) | 16.0 (7.0, 27.0) |  |  |
| Time from ICU admission to discharged alive (days) | 6.0 (2.0, 14.0) | 4.0 (2.0, 7.0) | 8.0 (4.0, 14.0) | 12.0 (5.0, 27.0) | 15.0 (7.0, 27.0) | 14.0 (7.0, 28.0) |
| HECTOR complication | 19 (9.6%) | 59 (10%) | 201 (31%) | 268 (1.7%) | 116 (6.2%) | 603 (16%) |
| Thrombosis | 11 (5.6%) | 53 (9.3%) | 121 (19%) | 172 (15%) | 50 (2.7%) | 586 (11%) |
| Hemorrhage | 9 (4.6%) | 6 (1.1%) | 97 (15%) | 123 (11%) | 78 (4.2%) | 19 (0.3%) |
| Discharged dead (HECTOR only) | 2 (11%) | 15 (25%) | 125 (62%) | 125 (47%) | 76 (66%) | 184 (31%) |
| Unadjusted OR for ICU mortality (HECTOR vs. non-HECTOR) | 1.20 (0.25, 5.66); p=0.819 | 0.64 (0.34, 1.22); p=0.179 | 1.59 (1.14, 2.23); p=0.007 | 1.51 (1.14, 1.99); p=0.004 | 22.7 (14.89, 34.46); p=<0.001 | 1.05 (0.87, 1.26); p=0.624 |
| OR for ICU mortality (HECTOR vs. non-HECTOR) adjusted for age, sex, ECMO | 0.77 (0.15, 3.96); p=0.750 | 0.60 (0.31, 1.16); p=0.130 | 1.43 (1.01, 2.02); p=0.045 | 1.46 (1.09, 1.96); p=0.012 | 12.88 (7.95, 20.87); p<0.001 | 0.88 (0.72, 1.08); p=0.226 |

**e-Table 6:** Sensitivity analysis of time-to-death/discharge using hazard ratios for ICU mortality estimated via Cox regression and via parametric Weibull regression and cumulative incidence sub-hazard ratios estimated via the Fine-Gray method. All models adjusted for: patient age, sex, BMI, any ECMO and country. The main survival analysis was performed via Cox regression (see Table 5). To check the validity of the estimates obtained via Cox regression the proportional hazards assumption was assessed (see e-Figure 3). The other survival methods presented in this Table (parametric Weibel regression model and Fine Gray model) provide sensitivity analyses with estimates that are broadly similar to the primary survival analysis. BMI body mass index; ECMO, extracorporeal membrane oxygenation. 95% confidence intervals given in parentheses.

|  | **Cox PH model with shared frailty** | **Fine-Gray competing risks model** | **Weibull parametric** |
| --- | --- | --- | --- |
| HECTOR | 1.01 (0.92, 1.12); p=0.784 | 1.14 (1.04, 1.26); p=0.008 | 1.05 (0.95, 1.15); p=0.339 |
| Age | 1.04 (1.03, 1.04); p<0.001 | 1.05 (1.04, 1.05); p<0.001 | 1.04 (1.04, 1.04); p<0.001 |
| Sex | 1.12 (1.03, 1.22); p=0.009 | 1.28 (1.17, 1.39); p<0.001 | 1.15 (1.06, 1.26); p=0.001 |
| BMI | 1.00 (0.99, 1.00); p=0.163 | 1.00 (0.99, 1.01); p=0.639 | 0.99 (0.99, 1.00); p=0.122 |
| ECMO | 1.20 (1.05, 1.37); p=0.006 | 2.53 (2.23, 2.86); p<0.001 | 1.14 (1.00, 1.30); p=0.056 |
| HECTOR - Hemorrhagic | 1.26 (1.09, 1.45); p=0.002 | 1.56 (1.34, 1.82); p<0.001 | 1.39 (1.20, 1.60); p<0.001 |
| Age | 1.04 (1.03, 1.04); p<0.001 | 1.05 (1.04, 1.05); p<0.001 | 1.04 (1.04, 1.04); p<0.001 |
| Sex | 1.12 (1.03, 1.22); p=0.008 | 1.28 (1.18, 1.40); p<0.001 | 1.16 (1.06, 1.26); p=0.001 |
| BMI | 1.00 (0.99, 1.00); p=0.180 | 1.00 (0.99, 1.01); p=0.652 | 0.99 (0.99, 1.00); p=0.135 |
| ECMO | 1.14 (1.00, 1.31); p=0.048 | 2.33 (2.05, 2.66); p<0.001 | 1.07 (0.94, 1.22); p=0.331 |
| HECTOR - Thrombotic | 0.88 (0.79, 0.99); p=0.030 | 0.96 (0.86, 1.07); p=0.465 | 0.88 (0.79, 0.99); p=0.030 |
| Age | 1.04 (1.03, 1.04); p<0.001 | 1.05 (1.04, 1.05); p<0.001 | 1.04 (1.04, 1.04); p<0.001 |
| Sex | 1.12 (1.03, 1.22); p=0.008 | 1.28 (1.18, 1.40); p<0.001 | 1.16 (1.06, 1.26); p=0.001 |
| BMI | 0.99 (0.99, 1.00); p=0.145 | 1.00 (0.99, 1.01); p=0.683 | 0.99 (0.99, 1.00); p=0.102 |
| ECMO | 1.21 (1.07, 1.38); p=0.003 | 2.63 (2.33, 2.98); p<0.001 | 1.15 (1.01, 1.31); p=0.030 |

**e-Table 7:** Sensitivity analyses of time-to-death/discharge using hazard ratios for ICU mortality estimated via Cox regression adjusted for: patient age, sex, any ECMO, country and vasopressor use. NB. inclusion of vasopressor data results in loss of 1,000 participants (>10% of sample) from the regression model. This confirms that inclusion of ‘vasopressor-use’ as a surrogate measure of disease severity was at least not associated with hazard for ICU mortality. Over-interpretation of this analysis should be avoided due to >10% loss of sample size from the regression model.

|  | **HR Adjusted for sex, age, BMI, ECMO** | **HR Adjusted for sex, age, BMI, ECMO, vasopressor use** |
| --- | --- | --- |
| HECTOR | 1.01 (0.92, 1.12); p=0.784 | 0.86 (0.78, 0.95); p=0.003 |
| HECTOR - Thrombosis | 0.88 (0.79, 0.99); p=0.030 | 0.80 (0.72, 0.90); p<0.001 |
| HECTOR - Hemorrhagic | 1.26 (1.09, 1.45); p=0.002 | 1.00 (0.87, 1.16); p=0.995 |

**e-Table 8:** Country-specific analysis of time-to-death/discharge for the 5 most represented countries using hazard ratios for ICU mortality estimated via Cox regression adjusted for: patient age, sex, BMI, ECMO (no shared frailty for country). NB. the country-specific estimates should be interpreted with caution since the sample sizes are lower than in the overall analysis. The comparison highlights the geographic variation seen within the database.

|  | **HECTOR** | | | | **HECTOR Thrombotic** | | | | **HECTOR Hemorrhagic** | | | |
| --- | --- | --- | --- | --- | --- | --- | --- | --- | --- | --- | --- | --- |
| **Country (n= sample size for adjusted model)** | **HR (95% CI)** | **P-value** | **Adj* HR (95% CI)** | **Adj* P-value** | **HR (95% CI)** | **P-value** | **Adj* HR (95% CI)** | **Adj* P-value** | **HR (95% CI)** | **P-value** | **Adj* HR (95% CI)** | **Adj* P-value** |
| **ALL** | **1.08 (0.99, 1.18)** | **0.067** | **1.01 (0.92, 1.12)** | **0.783** | **0.97 (1.87, 1.07)** | **0.519** | **0.88 (0.79, 0.99)** | **0.030** | **1.27 (1.13, 1.43)** | **<.001** | **1.26 (1.09, 1.45)** | **0.002** |
| Spain (n=4531) | 0.83 (0.71, 0.97) | 0.018 | 0.84 (0.71, 0.99) | 0.038 | 1.32 (0.71, 2.46) | 0.379 | 0.83 (0.70, 0.98) | 0.028 | 0.81 (0.69, 0.95) | 0.011 | 1.29 (0.68, 2.44) | 0.437 |
| Italy (n=1084) | 3.56 (2.65, 4.79) | <0.001 | 3.11 (2.06, 4.70) | <0.001 | 3.96 (2.89, 5.42) | <0.001 | 1.17 (0.71, 1.92) | 0.546 | 3.96 (2.89, 5.42) | <0.001 | 3.76 (2.45, 5.78) | <0.001 |
| USA (n=995) | 0.83 (0.66, 1.03) | 0.085 | 0.96 (0.77, 1.21) | 0.733 | 0.78 (0.59, 1.04) | 0.085 | 0.90 (0.69, 1.17) | 0.428 | 0.78 (0.59, 1.04) | 0.085 | 0.96 (0.71, 1.29) | 0.776 |
| Indonesia (n=473) | 1.20 (0.95, 1.51) | 0.121 | 1.18 (0.92, 1.53) | 0.189 | 1.27 (0.96, 1.68) | 0.091 | 0.93 (0.69, 1.26) | 0.639 | 1.27 (0.96, 1.68) | 0.091 | 1.44 (1.06, 1.96) | 0.019 |
| India (n=520) | 0.73 (0.39, 1.35) | 0.312 | 0.72 (0.38, 1.37) | 0.316 | 0.51 (0.13, 2.07) | 0.348 | 0.68 (034, 1.34) | 0.262 | 0.51 (0.13, 2.07) | 0.348 | 1.48 (0.20, 11.01) | 0.700 |
| Australia (n=134) | 0.47 (0.11, 2.08) | 0.322 | 0.51 (0.10, 2.57) | 0.413 | 2.03 (0.45, 9.13) | 0.356 | 3.00 (0.62, 14.54)** | 0.172 | 2.03 (0.45, 0.913) | 0.356 | 3.27 (0.67, 16.05) | 0.144 |

****adjusted only for age and BMI (not sex or ECMO) as there were only n=11 patients with thrombotic complications of which only 1 was female and only 1 received ECMO.

**e-Table 9:** Sensitivity analyses of time-to-death/discharge using hazard ratios for ICU mortality estimated via Cox regression and via parametric Weibull regression and cumulative incidence sub-hazard ratios estimated via the Fine-Gray method. All models adjusted for: patient age, sex, any ECMO, and country. NB. BMI not adjusted for due to high missing data. 95% confidence intervals given in parentheses. This provides a sensitivity analysis on whether BMI should be adjusted for as a covariate. Clinically, BMI may impact outcomes however this was not adjusted for as there was a substantial drop in sample size due to missing/invalid data in the BMI field (1000 patients). The findings presented here confirm the estimates are broadly similar to e-Table 6.

|  | **Cox PH model with shared frailty** | **Fine-Gray competing risks model** | **Weibull parametric** |
| --- | --- | --- | --- |
| HECTOR | 0.99 (0.91, 1.08); p=0.826 | 1.11 (1.02, 1.22); p=0.020 | 1.02 (0.94, 1.12); p=0.593 |
| Age | 1.04 (1.03, 1.04); p<0.001 | 1.05 (1.04, 1.05); p<0.001 | 1.04 (1.03, 1.04); p<0.001 |
| Sex | 1.13 (1.05, 1.22); p=0.002 | 1.30 (1.20, 1.41); p<0.001 | 1.16 (1.07, 1.25); p<0.001 |
| ECMO | 1.18 (1.05, 1.33); p=0.007 | 2.53 (2.26, 2.84); p<0.001 | 1.12 (0.99, 1.27); p=0.063 |
| HECTOR - Hemorrhagic | 1.22 (1.07, 1.40); p=0.003 | 1.52 (1.32, 1.76); p<0.001 | 1.35 (1.18, 1.54); p<0.001 |
| Age | 1.04 (1.03, 1.04); p<0.001 | 1.05 (1.04, 1.05); p<0.001 | 1.04 (1.03, 1.04); p<0.001 |
| Sex | 1.13 (1.05, 1.22); p=0.001 | 1.31 (1.21, 1.42); p<0.001 | 1.16 (1.07, 1.25); p<0.001 |
| ECMO | 1.13 (1.00, 1.28); p=0.050 | 2.36 (2.09, 2.65); p<0.001 | 1.06 (0.94, 1.20); p=0.331 |
| HECTOR - Thrombotic | 0.87 (0.78, 0.97); p=0.009 | 0.94 (0.85, 1.05); p=0.284 | 0.87 (0.79, 0.97); p=0.010 |
| Age | 1.04 (1.03, 1.04); p<0.001 | 1.05 (1.04, 1.05); p<0.001 | 1.04 (1.03, 1.04); p<0.001 |
| Sex | 1.13 (1.05, 1.23); p=0.001 | 1.31 (1.21, 1.42); p<0.001 | 1.16 (1.08, 1.26); p<0.001 |
| ECMO | 1.18 (1.05, 1.33); p=0.005 | 2.61 (2.33, 2.93); p<0.001 | 1.13 (1.01, 1.28); p=0.040 |

**e-Table 10:** Baseline patient characteristics with accompanying univariate analysis for patients requiring ECMO. SOFA, Sequential Organ Failure Assessment; APACHE, Acute Physiology and Chronic Health Evaluation.

|  | | **ECMO patients** | | | **No-ECMO patients** | | |
| --- | --- | --- | --- | --- | --- | --- | --- |
| **Characteristic** | **Class or Statistic** | **HECTOR (n=427)** | **Non-HECTOR (n=735)** | **P-value** | **HECTOR (n=1297)** | **Non-HECTOR (n=9108)** | **P-value** |
| Age (years) | median (Q1, Q3) | 53.0 (43.0, 60.0) | 50.0 (40.0, 58.0) | 0.0031 | 63.0 (55.0, 71.0) | 62.0 (52.0, 70.0) | 0.0036 |
| Body mass index (kg/m2) | median (Q1, Q3) | 30.4 (26.2, 33.9) | 29.9 (27.0, 34.9) | 0.6671 | 27.4 (24.2, 31.2) | 27.9 (25.0, 32.1) | <0.0001 |
| Sex | Female | 123 (29%) | 219 (30%) | 0.7210 | 384 (30%) | 2881 (32%) | 0.1448 |
|  | Male | 304 (71%) | 516 (70%) |  | 911 (70%) | 6218 (68%) |  |
| Ethnicity | White | 181 (48%) | 151 (27%) | <0.0001 | 175 (26%) | 1069 (32%) | 0.0026 |
|  | Black | 31 (8.2%) | 48 (8.7%) |  | 71 (11%) | 305 (9.2%) |  |
|  | Asian | 42 (11%) | 63 (11%) |  | 241 (36%) | 1044 (32%) |  |
|  | Hispanic, aboriginal | 57 (15%) | 215 (39%) |  | 96 (14%) | 404 (12%) |  |
|  | Other | 68 (18%) | 76 (14%) |  | 81 (12%) | 492 (15%) |  |
| Chronic cardiac disease | Yes | 34 (8.2%) | 26 (4.3%) | 0.0095 | 295 (23%) | 1287 (14%) | <0.0001 |
| Chronic kidney disease | Yes | 16 (3.9%) | 31 (5.1%) | 0.3381 | 159 (12%) | 705 (7.8%) | <0.0001 |
| Chronic neurological disorder | Yes | 10 (2.4%) | 14 (2.5%) | 0.9618 | 82 (6.4%) | 424 (5.4%) | 0.1477 |
| Chronic haematologic disorder | Yes | 11 (2.7%) | 19 (3.4%) | 0.5416 | 60 (4.7%) | 323 (4.1%) | 0.3580 |
| Diabetes | Yes | 99 (25%) | 103 (18%) | 0.0112 | 280 (25%) | 1450 (18%) | <0.0001 |
| Hypertension | Yes | 176 (42%) | 259 (43%) | 0.7954 | 705 (55%) | 5113 (57%) | 0.1204 |
| Smoking | Never smoked | 174 (43%) | 279 (48%) | 0.2180 | 600 (47%) | 4181 (53%) | <0.0001 |
|  | Current smoker | 74 (18%) | 94 (16%) |  | 376 (29%) | 2210 (28%) |  |
|  | Former smoker | 160 (39%) | 205 (35%) |  | 304 (24%) | 1536 (19%) |  |
| Malignant neoplasm | Yes | 6 (1.5%) | 7 (1.2%) | 0.7638 | 52 (4.0%) | 296 (3.8%) | 0.6264 |
| SOFA score | median (Q1, Q3) | 7.0 (5.0, 10.0) | 7.0 (4.0, 10.0) | 0.1026 | 5.0 (3.0, 9.0) | 4.0 (3.0, 7.0) | <0.0001 |
| APACHE II score | median (Q1, Q3) | 18.0 (12.0, 23.0) | 16.0 (10.0, 23.0) | 0.1964 | 16.0 (11.0, 24.0) | 14.0 (9.0, 19.0) | <0.0001 |

**e-Table 11:** Disposition outcomes for patients requiring ECMO

|  |  | **ECMO patients** | | | **No-ECMO patients** | | |
| --- | --- | --- | --- | --- | --- | --- | --- |
| **Characteristic** | **Class or Statistic** | **HECTOR (n=427)** | **Non-HECTOR (n=735)** | **P-value** | **HECTOR (n=1297)** | **Non-HECTOR (n=9108)** | **P-value** |
| Mortality at 28 days | Yes | 125 (30%) | 150 (21%) | 0.0006 | 296 (23%) | 1147 (13%) | <0.0001 |
| Mortality at 90days | Yes | 209 (50%) | 236 (33%) |  | 340 (26%) | 1246 (14%) | <0.0001 |
| Discharge disposition | Discharged dead | 240 (56%) | 340 (46%) | <0.0001 | 513 (40%) | 3139 (34%) | <0.0001 |
|  | Discharged alive | 101 (24%) | 289 (39%) |  | 670 (52%) | 5395 (59%) |  |
|  | Hospitalisation | 18 (4.2%) | 16 (2.2%) |  | 45 (3.5%) | 242 (2.7%) |  |
|  | Transferred to other facility | 67 (16%) | 89 (12%) |  | 65 (5.0%) | 313 (3.4%) |  |
|  | Palliative discharge | 1 (0.2%) | 1 (0.1%) |  | 4 (0.3%) | 19 (0.2%) |  |
| Time from ICU admission to death (days) | median (Q1, Q3) | 24.0 (14.0, 40.0) | 24.0 (14.0, 40.0) | 0.8794 | 9.5 (4.0, 20.0) | 9.0 (5.0, 17.0) | 0.9271 |
| Time from ICU admission to discharge alive (days) | median (Q1, Q3) | 41.0 (26.0, 57.0) | 33.0 (18.0, 53.0) | 0.0019 | 17.0 (8.0, 30.0) | 12.0 (6.0, 24.0) | <0.0001 |
| Time from admission to death (days) | median (Q1, Q3) | 24.0 (12.5, 38.5) | 24.0 (13.0, 42.0) | 0.5267 | 11.0 (5.0, 21.0) | 11.0 (6.0, 20.0) | 0.6819 |
| Time from admission to discharge alive (days) | median (Q1, Q3) | 50.5 (30.5, 74.0) | 40.0 (23.0, 63.0) | 0.0016 | 29.0 (17.0, 48.0) | 22.0 (13.0, 38.0) | <0.0001 |

**e-Table 12:** Cause of death for patients requiring ECMO with ICU mortality (by HECTOR)

|  | **ECMO patients** | | | **No-ECMO patients** | | |
| --- | --- | --- | --- | --- | --- | --- |
| **Cause of Death  n (column%)** | **Deaths**  **Non-HECTOR group (n=340)** | **Deaths HECTOR group (n=240)** | **Total deaths (n=580)** | **Deaths**  **Non-HECTOR group (n=3139)** | **Deaths HECTOR group (n=513)** | **Total deaths (n=3652)** |
| Respiratory failure | 72 (21%) | 59 (25%) | 131 | 1114 (35%) | 158 (31%) | 1272 |
| Multi-organ failure | 125 (37%) | 99 (29%) | 224 | 910 (29%) | 172 (34%) | 1082 |
| Unknown* | 46 (14%) | 6 (2.5%) | 52 | 634 (20%) | 20 (3.9%) | 654 |
| Septic shock | 28 (8.2%) | 24 (10%) | 52 | 196 (6.2%) | 50 (9.7%) | 246 |
| “Other” | 21 (6.2%) | 13 (5.4%) | 34 | 157 (5.0%) | 44 (8.6%) | 201 |
| Cardiac failure | 23 (6.8%) | 13 (5.4%) | 36 | 81 (2.6%) | 43 (8.4%) | 124 |
| Hemorrhagic shock | 8 (2.4%) | 7 (2.9%) | 15 | 23 (0.7%) | 4 (0.8%) | 27 |
| Cardiovascular accident | 1 (0.3%) | 2 (0.8%) | 3 | 20 (0.6%) | 9 (1.8%) | 29 |
| Cerebrovascular accident | 15 (4.4%) | 16 (6.7%) | 31 | 1 (<0.1%) | 11 (2.1%) | 12 |
| Liver failure | 1 (0.3%) | 1 (0.4%) | 2 | 2 (0.1%) | 2 (0.4%) | 4 |
| Treatment withdrawn, prognosis poor | 0 | 0 | 0 | 1 (<0.1%) | 0 | 1 |

*includes n=12 listed as “Not applicable”

**e-Table 13:** Data completeness

|  | **Data not entered (%)** | |
| --- | --- | --- |
| **Characteristic** | **HECTOR (n=1732)** | **Non-HECTOR (n=10237)** |
| Age | 0 (0%) | 0 (0%) |
| Body mass index (kg/m^2^) | 223 (13%) | 1505 (15%) |
| Sex | 2 (0.1%) | 9 (0.1%) |
| Ethnicity | 687 (40%) | 6337 (62%) |
| Chronic cardiac disease | 21 (1.2%) | 207 (2.0%) |
| Chronic kidney disease | 27 (1.6%) | 197 (1.9%) |
| Chronic neurological disorder | 27 (1.6%) | 1673 (16%) |
| Chronic hematologic disorder | 24 (1.4%) | 1672 (16%) |
| Diabetes mellitus | 203 (12%) | 1396 (14%) |
| Hypertension | 16 (0.9%) | 257 (2.5%) |
| Smoking | 36 (2.1%) | 1587 (16%) |
| Malignant neoplasm | 27 (1.6%) | 1675 (16%) |
| Baseline SOFA score | 1318 (76%) | 8781 (86%) |
| Baseline APACHE II score | 1299 (75%) | 8456 (83%) |
| Any invasive ventilation | 8 (0.5%) | 394 (3.8%) |
| Mechanical ventilation | 33 (1.9%) | 1028 (10%) |
| Mechanical ventilation (days)* | 399 (23%) | 3953 (39%) |
| Prone positioning (mechanical ventilation) | 620 (36%) | 5286 (52%) |
| Prone positioning (before ECMO)** | 87 (20%) | 363 (49%) |
| Inhaled nitric oxide | 614 (35%) | 5046 (49%) |
| Neuromuscular blockade (before ECMO)** | 89 (21%) | 345 (47%) |
| Tracheostomy | 38 (2.2%) | 1144 (11%) |
| ECMO | 8 (0.5%) | 394 (3.8%) |
| ECMO (days)* ** | 16 (3.7%) | 99 (13%) |
| Vasopressor use | 37 (2.1%) | 1978 (19%) |
| Anticoagulation therapy | 1234 (71%) | 8468 (83%) |
| ICU length of stay (days)* | 108 (6.2%) | 1335 (13%) |
| Hospital length of stay (days)* | 138 (8.0%) | 1552 (15%) |
| Time from admission to mechanical ventilation (days)* | 612 (35%) | 4136 (40%) |
| Time from admission to ECMO (days)* ** | 35 (8.2%) | 108 (15%) |
| Mortality at 28 days* | 20 (1.2%) | 102 (1.0%) |
| Mortality at 90 days* | 20 (1.2%) | 102 (1.0%) |

* includes invalid dates, e.g., due to data entry error.

** denominator only includes ECMO patients (HECTOR n=427, non-HECTOR n=735)

**e-Table 14:** country-specific data completeness for the 5 most represented countries in the dataset (and Australia). The comparison highlights the geographic variation seen within the database.

| **Characteristic** | **Australia** | **India** | **Indonesia** | **United States** | **Italy** | **Spain** |
| --- | --- | --- | --- | --- | --- | --- |
| **No. patients** | **198** | **570** | **644** | **1155** | **1876** | **5296** |
| **No. ECMO patients** | **15** | **6** | **22** | **296** | **141** | **141** |
| **No. IMV patients** | **100** | **140** | **348** | **852** | **1449** | **3796** |
| No. sites | 10 | 1 | 10 | 46 | 33 | 61 |
| Patients per site, min-max | 1-85 | 570 | 1-233 | 1-187 | 1-235 | 2-424 |
| Age | 0 | 0 | 0 | 0 | 0 | 0 |
| Body mass index (kg/m^2^) | 20 (10%) | 8 (1.4%) | 137 (21%) | 59 (5.1%) | 218 (12%) | 580 (11%) |
| Sex | 0 | 0 | 1 (0.2%) | 1 (0.1%) | 0 | 8 (0.2%) |
| Ethnicity | 40 (20%) | 1 (0.2%) | 6 (0.9%) | 57 (4.9%) | 1506 (80%) | 4988 (94%) |
| Chronic cardiac disease | 19 (9.6%) | 0 | 8 (1.2%) | 27 (2.3%) | 9 (0.5%) | 2 (<0.1%) |
| Chronic kidney disease | 19 (9.6%) | 0 | 12 (1.9%) | 24 (2.1%) | 7 (0.4%) | 2 (<0.1%) |
| Chronic neurological disorder | 20 (10%) | 1 (0.2%) | 8 (1.2%) | 29 (2.5%) | 1480 (79%) | 1 (<0.1%) |
| Chronic hematologic disorder | 19 (9.6%) | 0 | 7 (1.1%) | 31 (2.7%) | 1479 (79%) | 1 (<0.1%) |
| Diabetes mellitus | 19 (9.6%) | 4 (0.7%) | 13 (2.0%) | 27 (2.3%) | 12 (0.6%) | 1251 (24%) |
| Hypertension | 3 (1.5%) | 0 | 2 (0.3%) | 20 (1.7%) | 11 (0.6%) | 6 (0.1%) |
| Smoking | 19 (9.6%) | 0 | 0 | 5 (0.4%) | 1408 (75%) | 3 (0.1%) |
| Malignant neoplasm | 19 (9.6%) | 1 (0.2%) | 6 (0.9%) | 33 (2.9%) | 1478 (79%) | 5 (0.1%) |
| Baseline SOFA score | 110 (56%) | 570 (100%) | 528 (82%) | 735 (64%) | 1627 (87%) | 5143 (97%) |
| Baseline APACHE II score | 198 (100%) | 155 (27%) | 461 (72%) | 799 (69%) | 1660 (88%) | 5077 (96%) |
| Any invasive ventilation | 0 | 17 (3.0%) | 5 (0.8%) | 1 (0.1%) | 261 (14%) | 101 (1.9%) |
| Mechanical ventilation | 0 | 1 (0.2%) | 0 | 41 (3.6%) | 589 (31%) | 69 (1.3%) |
| Mechanical ventilation (days)* | 0 | 1 (0.7%) | 19 (5.5%) | 38 (4.5%) | 608 (42%) | 95 (2.5%) |
| Prone positioning (mechanical ventilation) | 100 (100%) | 0 | 2 (0.6%) | 48 (5.6%) | 52 (3.6%) | 3557 (94%) |
| Prone positioning (before ECMO)** | 9 (60%) | 1 (17%) | 13 (59%) | 63 (21%) | 42 (30%) | 129 (91%) |
| Inhaled nitric oxide | 198 (100%) | 1 (0.2%) | 2 (0.3%) | 58 (5.0%) | 249 (13%) | 4986 (94%) |
| Neuromuscular blockade (before ECMO)** | 10 (67%) | 1 (17%) | 12 (55%) | 62 (21%) | 24 (17%) | 129 (91%) |
| Tracheostomy | 198 (100%) | 1 (0.2%) | 1 (0.2%) | 43 (3.7%) | 596 (32%) | 96 (1.8%) |
| ECMO | 0 | 17 (3.0%) | 5 (0.8%) | 1 (0.1%) | 261 (14%) | 101 (1.9%) |
| ECMO (days)* ** | 15 (100%) | 0 | 0 | 20 (6.8%) | 37 (26%) | 28 (20%) |
| Vasopressor use | 198 (100%) | 1 (0.2%) | 1 (0.2%) | 48 (4.2%) | 1487 (79%) | 107 (2.0%) |
| Anticoagulation therapy | 198 (100%) | 2 (0.4%) | 499 (77%) | 903 (78%) | 1799 (96%) | 5154 (97%) |
| ICU length of stay (days)* | 42 (21%) | 20 (3.5%) | 44 (6.8%) | 86 (7.4%) | 651 (35%) | 195 (3.7%) |
| Hospital length of stay (days)* | 31 (16%) | 11 (1.9%) | 98 (15%) | 83 (7.2%) | 880 (47%) | 88 (1.7%) |
| Time from admission to mechanical ventilation (days)* | 5 (5.0%) | 6 (4.3%) | 46 (13%) | 197 (23%) | 492 (34%) | 24 (0.6%) |
| Time from admission to ECMO (days)* ** | 7 (47%) | 0 | 3 (14%) | 24 (8.1%) | 41 (29%) | 32 (23%) |
| Mortality at 28 days* | 3 (1.5%) | 11 (1.9%) | 9 (1.4%) | 14 (1.2%) | 7 (0.4%) | 1 (<0.1%) |
| Mortality at 90 days* | 3 (1.5%) | 11 (1.9%) | 9 (1.4%) | 14 (1.2%) | 7 (0.4%) | 1 (<0.1%) |

**e-Figures**

**e-Figure 1:** STROBE patient flow chart. Dashed lines/boxes represent the analyses for patients with recorded ECMO data.

**17,881** patients recorded in registry

**10,405** non-ECMO

**1,732 HECTOR**

**10,237 non-HECTOR**

**11,969** patients included in primary analysis

**11,567** patients included in ECMO

**5,909** patients excluded*

191 age <16 years

106 not admitted to ICU for coronavirus

98 admission date outside timeframe

2607 admission date not entered

3523 discharge disposition not entered

3 withdrawn

**402** ECMO data requirement not recorded

**1,162** ECMO

**7,35** non-HECTOR

**427** HECTOR

**9,108** non-HECTOR

**1,297** HECTOR

*****Subtypes of exclusions exceed total number of excluded patients as a patient could fail multiple inclusion criteria.

**e-Figure 2:** Kaplan-Meier survival curves for HECTOR subgroups. (A) patients who were known to not have a thrombotic complication (red) versus not (blue); (B) patients who were reported to have a hemorrhage complication (red) versus not (blue).

**A)**

**B)**

**e-Figure 3:** Log-log plot with HECTOR (red) and non-HECTOR (blue) groups. The Proportional Hazards assumption holds if the two lines are approximately parallel. Note: test of Schoenfeld residuals had p=0.100.


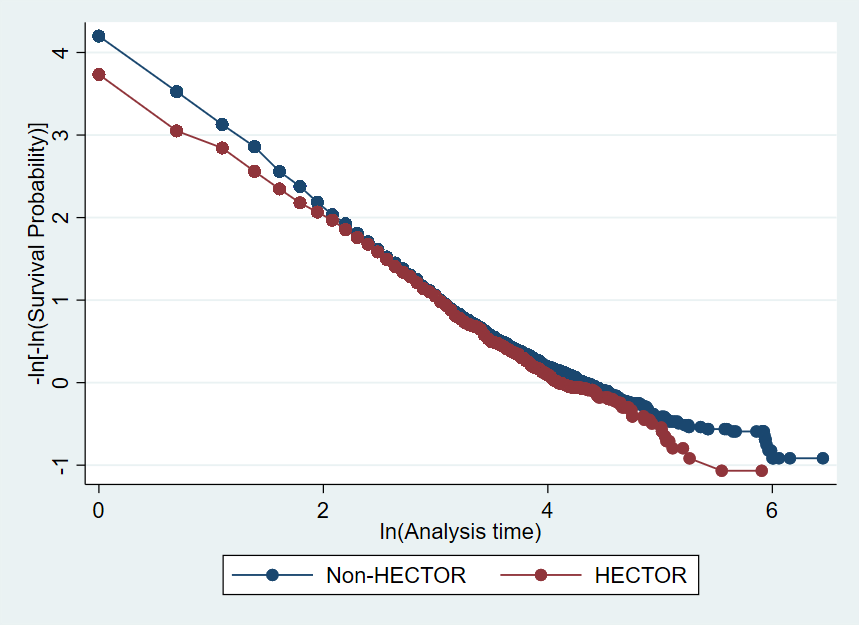


**e-Appendices**

**e-Appendix 1:** Full list of recruiting sites included in the CCCC and relevant IRB approvals

| **Site Name** | **Ethics Committee Name** | **Ethics Approval Number** |
| --- | --- | --- |
| Adult ICU Saiful Anwar Hospital | Health Research Ethics RSUD Dr. Saiful Anwar Malang | 400/131/K.3/302/2020 |
| Al-Adan Hospital | Kuwait Ministry of Health Ethics Committee | 1403/2020 |
| Allegheny General Hospital | Allegheny-Singer Research Institute (ASRI)-WPAHS Institutional Review Board | 2020-113 |
| Avera McKennan Hospital & University Health Center | Avera Institutional Review Board | IRB00001096 |
| Barmherzige Bruder Regensburg | Ethics Committee at the University of Regensburg | N/A |
| Baylor All Saints Medical Centre | Baylor Scott & White Research IRB | 345512 |
| Baylor Scott | Baylor Scott & White Research IRB | 345512 |
| Baylor University Medical Centre | Baylor Scott & White Research IRB | 345512 |
| Beth Israel Deaconess Medical Center | Beth Israel Deaconess Medical Center Committee on Clinical Investigations | 2020P000355 |
| Box Hill Hospital | The Alfred Ethics Committee | Alfred 108/20 |
| Carilion Clinic | Carilion Clinic Institutional Review Board | IRB-20-863 |
| Chiba University Hospital | Research Ethics Committees of Graduate School of Medicine, Chiba University | 3704 |
| Chonnam National University Hospital | Chonnam National University Hospital Institutional Review Board | CNUH-2020-070 |
| Civil Hospital Marie Curie, Brussels | Ethics Committee CHU-Charleroi | P20/27_24/03 (B3252020) |
| Clinica Pasteur de Neuquen (University of Comahue) | Teaching and Research Committee of the Clinical Pasteur | N/A |
| Cleveland Clinic - Florida | Cleveland Clinic Institutional Review Board | IRB#20-507 |
| Cleveland Clinic Abu Dhabi | Cleveland Clinic Abu Dhabi Research Ethics Committee | A-2020-086 |
| Cleveland Clinic - Ohio | Cleveland Clinic Institutional Review Board | IRB#20-507 |
| Clinica Alemana De Santiago | Scientific Ethics Committee, University of Development Center for Bioethics, Faculty of Medicine, Clinica Alemana de Santiago | 2020-25 |
| Clinica Las Condes, Chile | Ethics Committee of Clinica Las Condes | 2020P000355 |
| Clinica Valle del Lili | Biomedical Research Ethics Committee of Fundacion Valle del Lilli | 121-2020 |
| Dr Sardjito Government Hospital - Paediatric | Medical and Health Research Ethics Committee (MHREC), Dr Sardjito General Hospital | KE/FK/0787/EC/2020 |
| Fatmawati Hospital | Human Resources and Education Jakarta | DM 01.01/VIII.2/266/2020 |
| Fondazione IRCCS Policlinico of Milan (Fondazione IRCCS Ca' Granda Ospedale Maggiore Policlinico) | Milan Area 2 Ethics Committee | 7785390 |
| Fondazione Policlinico Universitario Agostino Gemelli IRCCS, Rome, Italy | Fondazione Policlinico Gemelli Ethics Committee | 3029 |
| Foothills Hospital | Conjoint Health Research Ethics Board, University of Calgary | REB20-0455 |
| Fujieda Municipal General Hospital | Ethics Committee of Fujieda Municipal General Hospital | No. 3 |
| Fukuoka University | Not Provided | H20-05-001 |
| Fundación Cardiovascular de Colombia | Scientific Technical Committee of the Cardiovascular Foundation of Colombia | No.164 of 2020 |
| Galway University Hospital | National Research Ethics Committee for COVID-19-related Health Research (NREC COVID-19), Ireland | 20-NREC-COV-017 |
| Geelong Hospital, Barwon Health | The Alfred Ethics Committee | Alfred 108/20 |
| Gold Coast Hospital | The Alfred Ethics Committee | Alfred 108/20 |
| Groote Schuur Hospital | Faculty of Health Sciences, Human Research Ethics Committee, University of Cape Town | 205/2020 |
| Hamad General Hospital | Medical Research Center, Hamad Medical Corporation, Doha, Qatar | MRC-05-006 |
| Harapan Kita National Heart Centre Hospital | Health Research Ethics of National Cardiac Center Harapan Kita Hospital | LB.02.01/VII/419/KEP. 026/2020 |
| Hartford HealthCare | Hartford HealthCare Human Research Protection Program Institutional Review Board | HHC-2020-0068 |
| Hiroshima University | Hiroshima University Epidemiological Research Ethics Board. | E1963 and 2.4.4 |
| Hospital Alemán | Independent Ethics Committee of Hospital Alemán CEIHA | N/A |
| Hospital Clinic, Barcelona | Clinical Research Ethics Committee Hospital Clinic Barcelona | HCB/2020/0370 |
| Hospital de Clinicas, Argentina | Ethics Committee of the Hospital de Clinicas | N/A |
| Hospital du Sacre Coeur | Ethics Committee for Research and Development of new technologies of the ICM, Montreal Heart Institute | Project Number: MP-33-2020-2776 |
| Hospital Emergencia Ate Vitarte | Ministry of Health Peru - Hospital Emergencia Ate Vitarte | 009-2020-ADI-HEAV |
| Hospital Nuestra Señora de Gracia Zaragoza | Clinical Research Ethics Committee Hospital Clinic Barcelona | HCB/2020/0370 |
| Hospital Universitari Sagrat Cor | Clinical Research Ethics Committee Hospital Group Quironsalud-Catalunya | 2020/65-UCI-HUSC |
| Hospital Universitario Sant Joan d'Alacant | Clinical Research Ethics Committee Hospital Clinic Barcelona | HCB/2020/0370 |
| Hospital Vall D Hebron | Research Ethics Committee with Medicines and Research Projects Committee of Hospital University Vall D'Hebron | 417 |
| Hospitales Puerta de Hierro, Mexico | Research Ethics Committee of Hospital Puera de Hierro | 3/30/2020 |
| Hospital Mount Sinai Medical Center | Institutional Review Board of Mount Sinai Medical Center | FWA00000176 |
| Hospital Verge de la Cintade Tortosa | Ethical Medical Research Committee of the Pere Virgili Institute of Health Research | 123/2020 |
| Houston Methodist Hospital | Houston Methodist Research Institute Institutional Review Board | PRO00026837 |
| Hyogo Prefectural Kakogawa Medical Center | Ethics Committee of Hyogo Prefectural Kakogawa Medical Center | R2-7 |
| INCOR (Universidade de São Paulo) | National Commission on Research Ethics, Brazil | 70.719-040 |
| INOVA Fairfax Medical Center, USA | Inova Health System Institutional Review Board | U20-03-3980 |
| Instituto Nacional Del Tórax | Eastern Metropolitan Health Service Scientific Ethics Committee. | N/A |
| ISMETT | IRCCS ISMETT sectional Ethics Committee | IRRB/09/20 |
| Johns Hopkins University | John Hopkins Medicine Institutional Review Board | IRB00247449 |
| Keimyung University Dong San Hospital | Keimyung University Dong San Medical Center | DSMC202004015-HE004 |
| Kimitsu Chuo Hospital | Not Provided | 526 |
| King Faisal Specialist Hospital and Research Center | Research Ethics Committee King Faisal Specialist Hospital and Research Centre | C380/800/41 |
| Klinik für Innere Medizin II | Ethics Committee University of Regensburg | 20-1747-101 |
| Klinikum Passau | Ethics Committee at the University of Regensburg | N/A |
| Kouritu Tousei Hospital | Ethics Committee of Kouritu Tousei Hospital | 885 |
| Kuwait ECLS program, Al-Amiri & Jaber Al-Ahmed Hospitals | Kuwait Ministry of Health Ethics Committee | 1405/2020 |
| Kyoto Medical Centre | Ethics Committee of Kyoto Medical Centre | 20-001 |
| Kyoto Prefectural University of Medicine | Ethics Committee of Kyoto Prefectural University of Medicine | ERB-C-1716 |
| Kyung Pook National University Chilgok Hospital | Kyung Pook National University Chilgok Hospital Institutional Review Board | KNUCH 2020-04-060 |
| Lancaster General Health | Lancaster General Hospital Institutional Review Board | 2020-24 |
| Lankenau Institute of Medical Research | Main Line Hospitals Institutional Review Board (MLH IRB) | E-20-5039 |
| Launceston Hospital | Tasmania Health and Medical Human Research Ethics Committee | 21666 |
| Legacy Emanuel Medical Center | Legacy Health Institutional Review Board | 1820 |
| London Health Sciences Centre | Western University Health Science Research Ethics Board | Project ID: 115907 |
| Maastricht University Medical Centre | Medical Ethics Review Committee, Maastricht University | METC2020-1566 |
| Manipal Hospital Whitefield | Ethics Committee of Manipal Hospitals, Bangalore | N/A |
| Mar del Plata Medical Foundation Private Community Hospital | Institutional Council of the Review of Research Studies (CIREI), Private Hospital of Community or Mar del Plata | 2919/2143/2020 |
| Mater Misericordiae University Hospital, Ireland | Mater Misericodiae University Hospital | 1/378/2137 |
| Mayo Clinic College of Medicine - Arizona | Mayo Clinic Institutional Review Board | 20-004851 |
| McGill University Health Centre | McGill University Health Centre Research Ethics Board | Project Number: 2020-6571 |
| Medical College of Wisconsin, USA | Medical College of Wisconsin Froedtert Hospital Institutional Review Board | PRO00037778 |
| Medical University of Vienna | Ethics Committee at Medical University of Vienna | EC Nr: 1276/2020 |
| Medizinische Klinik und Poliklinik II, Munich | Ethics Committee at the University of Regensburg | N/A |
| MedStar Washington Hospital Centre | Medstar Health Research Institute Institutional Review Board | STUDY00002284 |
| Nagoya University Hospital | Not Provided | 2020-0073-2 |
| National Taiwan University Hospital | Research Ethics Committee National Taiwan University Hospital | 202002009RINA |
| North Estonia Medical Centre, Tallin | Ethics Committee for Human Research of the Institute for Health Development (TAEIK) | Application Number 2121 |
| Northwell Health | Feinstein Institutes for Medical Research Northwell Health Institutional Review Board. | 25-0591 |
| Obihiro-Kosei General Hospital | Ethics Committee of Obihiro-Kosei General Hospital | 2020-069 |
| Ochsner Clinic Foundation | Ochsner Clinic Foundation Institutional Review Board | FWA00002050 |
| Ohio State University | The Ohio State Biomedical Sciences Institutional Review Board | 2020H0139 |
| Oklahoma Heart Institute | Hillscrest medical Center Institutional Review Board | N/A |
| Oregon Health and Science University Hospital | Oregon Health and Science University Institutional Review Board | STUDY00021357 |
| Ospedale Molinette Torino | Inter-Company Ethics Committee A.O.U City of Health and Science of Turin- A.O Maurizian Order of Turin- A.S.L City of Turin | 00103/2020 |
| Ospedale San Gerardo | Brianza Ethics Committee | N/A |
| Ospedale San Paolo | Milan Area 1 Ethics Committee | P/08/05/2020 |
| Pamela Youde Nethersole Eastern Hospital | Hong Kong East Cluster Research Ethics Committee (HKEC REC) | HKECREC-2020-0.16 |
| Penn Medicine | University of Pennsylvania Institutional Review Board | 842836 |
| Persahabatan Hospital | Ethic Committee of Health Research of Persahabatan Hospital | 61/KEPK-RSUPP/6/2020 |
| PICU Saiful Anwar Hospital | Health Research Ethics RSUD Dr. Saiful Anwar Malang | 400/131/K.3/302/2020 |
| Piedmont Atlanta Hospital, USA | Piedmont Healthcare Institutional Review Board | 1591828-1 |
| Policlinico di S. Orsola, Università di Bologna, Italy | Ethics Committee of the Vasta Emilia-Romagna Region | 1321/2020 |
| Policlinico of Padova, Padova | Ethics Committee for Clinical rials of the Province of Padua | 4851/A0/20 |
| Presbyterian Hospital Services | Presbyterian Healthcare Services Institutional Review Board | 1602073-1 |
| Princess Margaret Hospital, Hong Kong | Kowloon West Cluster Research Ethics Committee (KWC-REC) | KW/EX-20-051(154-10) |
| Prof Dr R. D. Kandou Central Hospital - Adult | Health Research Ethics Commission (KEPK) RSUP Prof Dr. RD Kandou | PP 04.03/XIX 2/126 12020 |
| Providence Saint John's Health Centre | Providence St Johns Health Institutional Review Board | STUDY2020000272 |
| Queen Elizabeth Hospital, Hong Kong | Research Ethics Committee (Kowloon Central/Kowloon East) | KC/KE-20-0064/ER-3 |
| Queen Mary Hospital the University of Hong Kong | University of Hong Kong/Hospital Authority Hong Kong West Cluster (HKU/HA HKW IRB) | UW 20-143 |
| Rinku General Medical Center | Ethics Committee of Rinku General Medical Center | 2020-006 |
| Rio Hortega University Hospital | Ethics Committee for the Clinical Research of the West-Valladolid Sanitary Area | PI077/20 |
| Rochester General Hospital | Rochester Regional Health Institutional Review Board | IRB 2018 B Hall |
| Royal Children's Hospital | The Alfred Ethics Committee | Alfred 108/20 |
| Royal North Shore Hospital | The Alfred Ethics Committee | Alfred 108/20 |
| Royal Prince Alfred Hospital | The Alfred Ethics Committee | Alfred 108/20 |
| RSPI Prof Dr Sulianti Saroso | Infectious Disease Hospital Prof. Dr Sulianti Saroso Committee of Health Research Ethics. | 09/XXXVIII.10/XI/2020 |
| RSUD Dr. Soetomo | Committee of Health Research Ethics RSUD Dr Soetomo Surabaya | 0096/KEPK/XI/2020 |
| Rush University | Rush University Medical Center Institutional Review Board | 20040706-IRB01 |
| Saiseikai Utsunomiya Hospital | Utsunomiya Hospital, Tochigi Prefecture | 2019 71 |
| Saiseikai Senri Hospital | Not provided | 20408 |
| San Martino Hospital | Reginal Ethics Committee of Liguria | 163/2020 |
| Sao Camilo Cura D'ars | Comitē de Ētica, Hospital Distrital Gonzaga Mota Messejana | N/A |
| Sāo Joāo Hospital Centre, Portugal | Ethics Committee for Health of the University Hospital Center of Sāo Joāo/Faculty of Medicine of the University of Porto | ID Number: 11002511 |
| Seoul National University Bundang Hospital | Seoul National University Bundang Hospital Institutional Review Board | B-2003/601-305 |
| Severance Hospital, Seoul | Yonsei University Health System, Severance Hospital, Institutional Review Board | 4-2020-0099 |
| Sinai Health Systems | University Health Network Research Ethics Board | Project ID: 2129 |
| Siriraj Hospital | Siriraj Institutional Review Board | 230/2563(IRB3) |
| Sozialmedizinisches Zentrum Süd - Kaiser-Franz-Josef-Spital | Ethics Committee of the City of Vienna | EK 20-064-VK |
| St Christopher's Hospital for Children | Drexel University Institutional Review Board | 2005007860 |
| St George Hospital | The Alfred Ethics Committee | Alfred 108/20 |
| St Vincent's Hospital Sydney | The Alfred Ethics Committee | Alfred 108/20 |
| St. Boniface Hospital, Manitoba | University of Manitoba Health Research Board | HS23821 |
| Tartu University Hospital, Tartu | Ethics Committee for Human Research of the Institute for Health Development (TAEIK) | Application Number 2121 |
| Teine Keijinkai Hospital | The Institutional Review Board at Teine Keijinkai Hospital | 2-019180-00 |
| The Alfred Hospital | The Alfred Ethics Committee | Alfred 108/20 |
| The Heart Hospital Baylor Plano | Baylor Scott & White Research IRB | 345512 |
| The University of Utah | Institutional Review Board the University of Utah | IRB_00131842 |
| Tohoku Medical and Pharmaceutical University | Not Provided | 2020-2-011 |
| Tokyo Metropolitan Tama Medical Center | Ethics Committee of Tokyo Metropolitan Tama Medical Center | 72 |
| Tufts Medical Centre | Tufts Health Sciences Institutional Review Board | MOD-01-STUDY00000808 |
| UH Cleveland Hospital | University Hospitals Institutional Review Board | STUDY20200428 |
| Uniklinik (University Hospital Frankfurt) | Ethics Committee of the Faculty of Medicine at Goethe University | 20-668 |
| University Airlangga Hospital (Paediatric) | Airlangga University Hospital Research Committee. | 178-A/KEP/2020 |
| University Hospital in Krakow | Bioethical Commission, The University Jagiellonian | 1072.6120.86.2020 |
| University of California San Francisco - Fresno | Community Medical Centers Institutional Review Board | 2020021 |
| University of Chicago | The University of Chicago Biological Sciences Division Institutional Review Board | IRB20-0579 |
| University of Cincinnati | University of Cincinnati Institutional Review Board | 2020-0406 |
| University of Iowa | University of Iowa Institutional Review Board | 202004380 |
| University of Nebraska Medical Center | University of Nebraska Medical Centre Institutional Review Board | 233-20-EP |
| University of Alabama at Birmingham Hospital | University of Alabama Institutional Review Board | IRB-300005234 |
| University of Florida | University of Florida Institutional Review Board | IRB202000933 |
| University of Oklahoma Health Sciences Center | University of Oklahoma Institutional Review Board for the Protection of Human Subjects | 11978 |
| UT Southwestern | University of Texas Southwestern Medical Center Institutional Review Board | N/A |
| Washington University in St. Louis, USA | The Washington University in St. Louis Institutional Review Board | 202004038 |
| Yokohama City University Medical Center | Ethics Committee of Yokohama City University Medical Center | B200500022 |
| Hospital Clinic De Barcelona | These sites received approvals to participate in the CIBERESUCICOVID study. CIBERSUCICOVID and COVID Critical executed a data sharing agreement to merge data into COVID Critical. Hospital Clinic Barcelona is considered the data controller for CIBERESUCICOVID so therefore ethics was approved by Clinical Research Ethics Committee, Hospital Clinic of Barcelona. | Ethics approval number HCB/2020/0370 |
| Hospital Universitario Sant Joan DAlacant |  |  |
| HULA |  |  |
| Hospital Nuestra Senora De Gracia |  |  |
| Hospital De Bellvitge |  |  |
| Clinica Sagrada Familia |  |  |
| Hospital Vall D’Hebron |  |  |
| Hospital Clinico Universitario De Valladolid |  |  |
| Hospital Universitario De Leon |  |  |
| Hospital Arnau De Vilanova De Lleida |  |  |
| Hospital San Pedro De Alcantara |  |  |
| Hospital Sagrat Cor |  |  |
| HUIL |  |  |
| Hospital La Fe De Valencia |  |  |
| Hospital Universitari Mutua Terrassa |  |  |
| Hospital Del Mar |  |  |
| Hospital Universitario Central De Asturias |  |  |
| Hospital De Mataro |  |  |
| Hospital Universitario De Valme |  |  |
| CHUO |  |  |
| Caupa |  |  |
| Hospital La Paz |  |  |
| Hospital Alvaro Cunqueiro |  |  |
| Hospital Universitario De Salamanca |  |  |
| Tortosa |  |  |
| Hospital Clinic Universitari De Valencia |  |  |
| Husll |  |  |
| Hospital Santa Maria De Lleida |  |  |
| Hospital Jerez De La Frontera |  |  |
| Hospital Universitario San Agustin |  |  |
| Parc Tauli |  |  |
| Hospital Clinico Universitario De Santiago |  |  |
| HUMV |  |  |
| Hospital Germans Trias I Pujol |  |  |
| Hospital De Torrejon |  |  |
| H.U.Basurto |  |  |
| Hospital Universitari Joan XXIII De Tarragona |  |  |
| Hospital Universitario De Cruces |  |  |
| Hospital De La Princesa |  |  |
| Hospital Universitario Reina Sofia (Hurs) |  |  |
| Hugc Dr Negrin |  |  |
| Hospital Son Espases |  |  |
| Hospital Universitario Hm Sanchinarro |  |  |
| Hospital Universitario Virgen Del Rocio |  |  |
| Hospital Virgen Macarena |  |  |
| Hospital San Juan De Dios Bormujos Aljarafe |  |  |
| Hospital General Universitario Gregorio Marano |  |  |
| Hospital De Getafe |  |  |
| Hospital Ramon Y Cajal |  |  |
| Hospital Universitario Rio Hortega |  |  |
| Hospital Universitario Principe De Asturias |  |  |
| HPE |  |  |
| Hospital 12 De Octubre |  |  |
| Hospital De Mostoles |  |  |
| Policlinico di Milano | On behalf of these sites, as data controller for the Italian sites, Policlinico of Milan executed a data sharing agreement with the COVID Critical study. Ethics was approved by the Milan Area 2 Ethics Committee. | Ethics approval number 7785390 |
| Niguarda |  |  |
| San Gerardo |  |  |
| Mantova Poma |  |  |
| Varese Sette Laghi |  |  |
| Humanitas Research Hospital |  |  |
| San Giovanni Molinette |  |  |
| Sant'Orsola Bologna |  |  |
| ASST Lecco - Ospedale di Merate |  |  |
| ASST Lecco - Ospedale A. Manzoni |  |  |
| ASST Nord Milano - Ospedale Edoardo Bassini - Cinisello Balsamo |  |  |
| ASST-MONZA, Ospedale di Desio |  |  |
| AULSS 5 Polesana - Ospedale di Rovigo e Ospedale di Trecenta |  |  |
| AULSS 9 Scaligera - Ospedale Magalini di Villafranca |  |  |
| Azienda Ospedaliera di Perugia |  |  |
| Azienda Sanitaria Universitaria Friuli Centrale - Udine |  |  |
| Azienda Ospedaliero - Universitaria di Modena |  |  |
| Policlinico Universitario Fondazione Agostino Gemelli - Roma |  |  |
| Azienda Ospedaliera Mater Domini - Catanzaro |  |  |
| Azienda Ospedaliera Universitaria Federico II |  |  |
| Azienda Ospedaliero-universitaria di Ferrara |  |  |
| Azienda Ospedaliera Universitaria Foggia - Ospedali Riuniti |  |  |
| AOU - Ospedali Riuniti di Ancona |  |  |
| Policlinico Universitario Paolo Giaccone - Palermo |  |  |

**e-Appendix 2:** List of contributors (to be listed in PubMed)

| **Prefix/First Name/Last Name** | **Site Name** |
| --- | --- |
| Tala Al-Dabbous Huda Alfoudri Mohammed Shamsah | Al Adan Hospital |
| Subbarao Elapavaluru Ashley Berg Christina Horn | Allegheny General Hospital |
| Yunis Mayasi | Avera McKennan Hospital & University Health Centre |
| Stephan Schroll | Barmherzige Bruder Regansburg |
| Dan Meyer Jorge Velazco Ludmyla Ploskanych Wanda Fikes Rohini Bagewadi Marvin Dao Haley White  Alondra Berrios Laviena  Ashley Ehlers Maysoon Shalabi-McGuire Trent Witt | Baylor Scott & White Health |
| Lorenzo Grazioli Luca Lorini | Bergamo Hospital |
| E. Wilson Grandin Jose Nunez Tiago Reyes | Beth Israel Deaconess Medical Centre |
| Diarmuid O’Briain Stephanie Hunter | Box Hill Hospital |
| Mahesh Ramanan Julia Affleck | Caboolture Hospital |
| Hemanth Hurkadli Veerendra  Sumeet Rai Josie Russell-Brown Mary Nourse | Canberra Hospital |
| Mark Joseph Brook Mitchell Martha Tenzer | Carilion Clinic |
| Ryuzo Abe | Chiba University Graduate School of Medicine |
| Hwa Jin Cho In Seok Jeong | Chonnam National University Hospital |
| Nadeem Rahman Vivek Kakar | Cleveland Clinic- Abu Dhabi |
| Nicolas Brozzi | Cleveland Clinic - Florida |
| Omar Mehkri Sudhir Krishnan Abhijit Duggal Stuart Houltham | Cleveland Clinic - Ohio |
| Jerónimo Graf | Clinica Alemana De Santiago |
| Roderigo Diaz Roderigo Orrego  Camila Delgado Joyce González Maria Soledad Sanchez Michael Piagnerelli Josefa Valenzuela Sarrazin | Clinica Las Condez |
| A/Prof. Gustavo Zabert Lucio Espinosa Paulo Delgado Victoria Delgado | Clinica Pasteur National- University of Comahue |
| Diego Fernando Bautista Rincón Angela Maria Marulanda Yanten Melissa Bustamante Duque | Clinica Valle de Lilli |
| Daniel Brodie | Medical ICU, Columbia College of Physicians and Surgeons, New-York-Presbyterian Hospital, NY, NY, USA |
| Alyaa Elhazmi Abdullah Al-Hudaib | Dr Sulaiman Alhabib Medical Group – Research Center, Riyadh, Saudi Arabia |
| Maria Callahan | Emory University Healthcare System |
| M. Azhari Taufik  Elizabeth Yasmin Wardoyo Margaretha Gunawan Nurindah S Trisnaningrum Vera Irawany Muhammad Rayhan | Fatmawati Hospital |
| Mauro Panigada Antonio Pesenti Alberto Zanella Giacomo Grasselli Sebastiano Colombo  Chiara Martinet  Gaetano Florio | Fondazione IRCCS Policlinico of Milan (Fondazione IRCCS Ca' Granda Ospedale Maggiore Policlinico) |
| Massimo Antonelli Simone Carelli Domenico L. Grieco | Fondazione Policlinico Universitario Agostino Gemelli IRCCS |
| Motohiro Asaki | Fujieda Municipal General Hospital |
| Kota Hoshino | Fukuoka University |
| Leonardo Salazar  Mary Alejandra Mendoza Monsalve | Fundación Cardiovascular de Colombia |
| John Laffey Bairbre McNicholas David Cosgrave | Galway University Hospitals |
| Joseph McCaffrey Allison Bone | Geelong Hospital |
| Yusuff Hakeem | Glenfield Hospital |
| James Winearls Mandy Tallott | Gold Coast University Hospital |
| David Thomson Christel Arnold-Day Jerome Cupido Zainap Fanie Malcom Miller Lisa Seymore Dawid van Straaten | Groote Schuur Hospital |
| Ali Ait Hssain Jeffrey Aliudin Al-Reem Alqahtani Khoulod Mohamed Ahmed Mohamed Darwin Tan Joy Villanueva Ahmed Zaqout | Hamad General Hospital - Weill Cornell Medical College in Qatar |
| Ethan Kurtzman Arben Ademi Ana Dobrita Khadija El Aoudi Juliet Segura | Hartford HealthCare |
| Gezy Giwangkancana | Hasan Sadikin Hospital (Adult) |
| Shinichiro Ohshimo | Hiroshima University |
| Javier Osatnik | Hospital Alemán |
| Anne Joosten | Hospital Civil Marie Curie |
| Antoni Torres Minlan Yang Ana Motos | Hospital Clinic, Barcelona |
| Carlos Luna | Hospital de Clínicas |
| Francisco Arancibia | Hospital del Tórax |
| Virginie Williams Alexandre Noel | Hospital du Sacre Coeur (Universite de Montreal) |
| Nestor Luque | Hospital Emergencia Ate Vitarte |
| Marina Fantini | Hospital Mater Dei |
| Ruth Noemi Jorge García Enrique Chicote Alvarez | Hospital Nuestra Señora de Gracia |
| Anna Greti | Hospital Puerta de Hierro |
| Adrian Ceccato | Hospital Universitari Sagrat Cor |
| Angel Sanchez | Hospital Universitario Sant Joan d’Alacant |
| Ana Loza Vazquez | Hospital Universitario Virgen de Valme |
| Ferran Roche-Campo Diego Franch-Llasat | Hospital Verge de la Cinta de Tortosa |
| Divina Tuazon | Houston Methodist Hospital |
| Marcelo Amato Luciana Cassimiro Flavio Pola Francis Ribeiro Guilherme Fonseca | INCOR (Universidade de São Paulo) |
| Heidi Dalton Mehul Desai Erik Osborn Hala Deeb | INOVA Fairfax Hospital |
| Antonio Arcadipane Gennaro Martucci Giovanna Panarello Chiara Vitiello Claudia Bianco Giovanna Occhipinti Matteo Rossetti Raffaele Cuffaro | ISMETT |
| Sung-Min Cho  Glenn Whitman | Johns Hopkins |
| Hiroaki Shimizu Naoki Moriyama | Kakogawa Acute Care Medical Center |
| Jae-Burm Kim | Keimyung University Dong San Hospital |
| Nobuya Kitamura | Kimitsu Chuo Hospital |
| Johannes Gebauer | Klinikum Passau |
| Toshiki Yokoyama | Kouritu Tousei Hospital |
| Abdulrahman Al-Fares Sarah Buabbas Esam Alamad Fatma Alawadhi Kalthoum Alawadi | Al-Amiri and Jaber Al-Ahmed Hospitals, Kuwait Extracorporeal Life Support Program |
| Hiro Tanaka | Kyoto Medical Centre |
| Satoru Hashimoto Masaki Yamazaki | Kyoto Prefectural University of Medicine |
| Tak-Hyuck Oh | Kyung Pook National University Chilgok Hospital |
| Mark Epler Cathleen Forney  Louise Kruse Jared Feister Joelle Williamson Katherine Grobengieser | Lancaster General Health |
| Eric Gnall Sasha Golden Mara Caroline  Timothy Shapiro Colleen Karaj Lisa Thome Lynn Sher Mark Vanderland Mary Welch Sherry McDermott | Lankenau Institute of Medical Research (Main Line Health) |
| Matthew Brain Sarah Mineall | Launceston General Hospital |
| Dai Kimura | Le Bonheur Children’s Hospital |
| Luca Brazzi Gabriele Sales  Giorgia Montrucchio | Le Molinette Hospital (Ospedale Molinette Torino) |
| Tawnya Ogston | Legacy Emanuel Medical Center |
| Dave Nagpal Karlee Fischer | London Health Sciences Centre |
| Roberto Lorusso | Maastricht University Medical Centre |
| Rajavardhan Rangappa Sujin Rai  Argin Appu | Manipal Hospital Whitefield |
| Mariano Esperatti  Nora Angélica Fuentes  Maria Eugenia Gonzalez | Hospital Privado de Comunidad. Mar del Plata. Escuela Superior de Medicina. Universidad Nacional de Mar del Plata |
| Diarmuid O’Briain | Maroondah Hospital |
| Edmund G. Carton | Mater Misericordiae University Hospital |
| Ayan Sen Amanda Palacios Deborah Rainey | Mayo Clinic College of Medicine |
| Gordan Samoukoviv Josie Campisi | McGill University Health Centre |
| Lucia Durham Emily Neumann Cassandra Seefeldt Octavio Falcucci Amanda Emmrich Jennifer Guy Carling Johns Kelly Potzner Catherine Zimmermann Angelia Espinal | Medical College of Wisconsin (Froedtert Hospital) |
| Nina Buchtele Michael Schwameis  Andrea Korhnfehl  Roman Brock  Thomas Staudinger | Medical University of Vienna |
| Stephanie-Susanne Stecher Michaela Barnikel Sófia Antón  Alexandra Pawlikowski | Medical Department II, LMU Hospital Munich |
| Akram Zaaqoq Lan Anh Galloway Caitlin Merley | MedStar Washington Hospital Centre |
| Alistair Nichol | Monash University |
| Marc Csete Luisa Quesada Isabela Saba | Mount Sinai Medical Centre |
| Daisuke Kasugai Hiroaki Hiraiwa Taku Tanaka | Nagoya University Hospital |
| Eva Marwali Yoel Purnama Santi Rahayu Dewayanti Ardiyan Dafsah Arifa Juzar Debby Siagian | National Cardiovascular Center Harapan Kita, Jakarta, Indonesia |
| Yih-Sharng Chen | National Taiwan University Hospital |
| Mark Ogino | Nemours Alfred I duPont Hospital for Children |
| Indrek Ratsep Andra-Maris Post Piret Sillaots  Anneli Krund  Merili-Helen Lehiste  Tanel Lepik | North Estonia Medical Centre |
| Frank Manetta Effe Mihelis Iam Claire Sarmiento Mangala Narasimhan Michael Varrone | Northwell Health |
| Mamoru Komats | Obihiro-Kosei General Hospital |
| Julia Garcia-Diaz Catherine Harmon | Ochsner Clinic Foundation |
| S. Veena Satyapriya Amar Bhatt Nahush A. Mokadam Alberto Uribe Alicia Gonzalez Haixia Shi Johnny McKeown Joshua Pasek Juan Fiorda Marco Echeverria | Ohio State University Medical Centre |
| Rita Moreno | Oklahoma Heart Institute |
| Bishoy Zakhary | Oregon Health and Science University Hospital (OHSU) |
| Marco Cavana Alberto Cucino | Ospedale di Arco (Trento Hospital) |
| Giuseppe Foti Marco Giani Benedetta Fumagalli | Ospedale San Gerardo |
| Davide Chiumello Valentina Castagna | Ospedale San Paolo |
| Andrea Dell’Amore Paolo Navalesi | Padua University Hospital (Policlinico of Padova) |
| Hoi-Ping Shum | Pamela Youde Nethersole Eastern Hospital |
| Alain Vuysteke | Papworth Hospitals NHS Foundation Trust |
| Asad Usman Andrew Acker Benjamin Smood Blake Mergler Federico Sertic Madhu Subramanian Alexandra Sperry Nicolas Rizer | Penn Medicine (Hospital of the University of Pennsylvania) |
| Erlina Burhan  Menaldi Rasmin Ernita Akmal Faya Sitompul Navy Lolong Bhat Naivedh | Persahabatan General Hospital |
| Simon Erickson | Perth Children's Hospital |
| Peter Barrett David Dean Julia Daugherty | Piedmont Atlanta Hospital |
| Antonio Loforte | Policlinico di S. Orsola, Università di Bologna |
| Irfan Khan Mohammed Abraar Quraishi Olivia DeSantis | Presbyterian Hospital Services, Albuquerque |
| Dominic So Darshana Kandamby | Princess Margaret Hospital |
| Jose M. Mandei Hans Natanael | Prof Dr R. D. Kandou General Hospital - Paediatric |
| Eka YudhaLantang Anastasia Lantang | Prof Dr R. D R. D. Kandou General Hospital - Adult |
| Surya Oto Wijaya | Dr Sulianti Saroso Hospital |
| Anna Jung | Providence Saint John's Health Centre |
| George Ng Wing Yiu Ng | Queen Elizabeth Hospital, Hong Kong |
| Pauline Yeung Ng  Shu Fang | The University of Hong Kong |
| Alexis Tabah Megan Ratcliffe Maree Duroux | Redcliffe Hospital |
| Shingo Adachi Shota Nakao | Rinku General Medical Center (and Senshu Trauma and Critical Care Center) |
| Pablo Blanco Ana Prieto Jesús Sánchez | Rio Hortega University Hospital |
| Meghan Nicholson | Rochester General Hospital |
| Warwick Butt Alyssa Serratore Carmel Delzoppo | Royal Children’s Hospital |
| Pierre Janin Elizabeth Yarad | Royal North Shore Hospital |
| Richard Totaro Jennifer Coles | Royal Prince Alfred Hospital |
| Bambang Pujo | RSUD Soetomo |
| Robert Balk Andy Vissing Esha Kapania James Hays Samuel Fox Garrett Yantosh Pavel Mishin | Rush University, Chicago |
| Saptadi Yuliarto Kohar Hari Santoso Susanthy Djajalaksana | Saiful Anwar Malang Hospital (Brawijaya University) (Paediatrics) |
| Arie Zainul Fatoni | Saiful Anwar Malang Hospital (Brawijaya University) (Adult) |
| Masahiro Fukuda | Saiseikai Senri Hospital |
| Keibun Liu | Saiseikai Utsunomiya Hospital |
| Paolo Pelosi Denise Battaglini | San Martino Hospital |
| Juan Fernando Masa Jiménez | San Pedro de Alcantara Hospital |
| Diego Bastos | Sao Camilo Cura D’ars |
| Sérgio Gaião | São João Hospital Centre, Porto |
| Desy Rusmawatiningtyas | Sardjito Hospital (Paediatrics) |
| Young-Jae Cho | Seoul National University Bundang Hospital |
| Su Hwan Lee | Severance Hospital |
| Tatsuya Kawasaki | Shizuoka Children’s Hospital |
| Laveena Munshi | Sinai Health Systems (Mount Sinai Hospital) |
| Pranya Sakiyalak Prompak Nitayavardhana | Siriraj Hospital |
| Tamara Seitz | Sozialmedizinisches Zentrum Süd – Kaiser-Franz-Josef-Spital |
| Rakesh Arora David Kent | St Boniface Hospital (University of Mannitoba) |
| Daniel Marino | St Christopher’s Hospital for Children |
| Swapnil Parwar Andrew Cheng Jennene Miller | St George Hospital |
| Shigeki Fujitani Naoki Shimizu | St Marianna Medical University Hospital |
| Jai Madhok Clark Owyang | Stanford University Hospital |
| Hergen Buscher Claire Reynolds | St Vincent’s Hospital |
| Olavi Maasikas AleksanBeljantsev Vladislav Mihnovits | Tartu University Hospital |
| Takako Akimoto Mariko Aizawa Kanako Horibe Ryota Onodera | Teine Keijinkai Hospital |
| Carol Hodgson Aidan Burrell Meredith Young | The Alfred Hospital |
| Timothy George | The Heart Hospital Baylor Plano, Plano |
| Kiran Shekar  Niki McGuinness Lacey Irvine | The Prince Charles Hospital |
| Brigid Flynn | The University of Kansas Medical Centre |
| Tomoyuki Endo | Tohoku Medical and Pharmaceutical University |
| Kazuhiro Sugiyama | Tokyo Metropolitan Bokutoh Hospital |
| Keiki Shimizu | Tokyo Metropolitan Medical Center |
| Eddy Fan Kathleen Exconde | Toronto General Hospital |
| Shingo Ichiba | Tokyo Women’s Medical University Hospital |
| Leslie Lussier | Tufts Medical Centre (and Floating Hospital for Children) |
| Gösta Lotz | Universitätsklinikum Frankfurt (University Hospital Frankfurt) (Uniklinik) |
| Maximilian Malfertheiner Lars Maier Esther Dreier | Universitätsklinikum Regensburg (Klinik für Innere Medizin II) |
| Neurinda Permata Kusumastuti | University Airlangga Hospital (Paediatric) |
| Colin McCloskey Al-Awwab Dabaliz Tarek B Elshazly Josiah Smith | University Hospital Cleveland Medical Centre (UH Cleveland Hospital) |
| Konstanty S. Szuldrzynski Piotr Bielański | University Hospital in Krakow |
| Yusuff Hakeem | University Hospitals of Leicester NHS Trust (Glenfield Hospital) |
| Keith Wille | University of Alabama at Birmingham Hospital (UAB) |
| Srinivas Murthy | University of British Columbia |
| Ken Kuljit S. Parhar Kirsten M. Fiest  Cassidy Codan Anmol Shahid | University of Calgary (Peter Lougheed Centre, Foothills Medical Centre, South Health Campus and Rockyview General Hospital) |
| Mohamed Fayed Timothy Evans Rebekah Garcia Ashley Gutierrez Hiroaki Shimizu | University of California, San Francisco-Fresno Clinical Research Centre |
| Tae Song Rebecca Rose | University of Chicago |
| Suzanne Bennett Denise Richardson | University of Cincinnati Medical Centre |
| Giles Peek | University of Florida |
| Lovkesh Arora Kristina Rappapport Kristina Rudolph Zita Sibenaller Lori Stout Alicia Walter | University of Iowa |
| Daniel Herr Nazli Vedadi | University of Maryland - Baltimore |
| Robert Bartlett | University of Michigan Medical Center |
| Antonio Pesenti | University of Milan |
| Shaun Thompson    Julie Hoffman Xiaonan Ying | University of Nebraska Medical Centre |
| Ryan Kennedy | University of Oklahoma Health Sciences Centre (OU) |
| Muhammed Elhadi | Faculty of Medicine, University of Tripoli |
| Matthew Griffee Anna Ciullo Yuri Kida | University of Utah Hospital |
| Ricard Ferrer Roca JordI Riera Sofia Contreras Cynthia Alegre | Vall d'Hebron University Hospital, Barcelona |
| Christy Kay Irene Fischer Elizabeth Renner | Washington University in St. Louis/ Barnes Jewish Hospital |
| Hayato Taniguci | Yokohama City University Medical Center |
| John Fraser Gianluigi Li Bassi Jacky Suen Adrian Barnett Nicole White Kristen Gibbons Simon Forsyth Amanda Corley  India Pearse Samuel Hinton Gabriella Abbate Halah Hassan Silver Heinsar Varun A Karnik Katrina Ki Hollier F. O'Neill Nchafatso Obonyo Leticia Pretti Pimenta Janice D. Reid Kei Sato Kiran Shekar Aapeli Vuorinen Karin S. Wildi Emily S. Wilson Stephanie Yerkovich | COVID-19 Critical Care Consortium |
| James Lee Daniel Plotkin Barbara Wanjiru Citarella Laura Merson | ISARIC, Centre for Tropical Medicine and Global Health, University of Oxford, Oxford, UK |

**e-Appendix 3**: List of collaborators

| **Prefix/First Name/Last Name** | **Site Name** |
| --- | --- |
| Emma Hartley | Aberdeen Royal Infirmary (Foresterhill Health Campus) |
| Bastian Lubis | Adam Malik Hospital |
| Takanari Ikeyama | Aichi Childrens Health and Medical Center |
| Balu Bhaskar | American Hospital |
| Jae-Seung Jung | Anam Korea University Hospital |
| Shay McGuinness | Auckland City Hospital |
| Glenn Eastwood | Austin Hospital |
| Sandra Rossi Marta  Fabio Guarracino | Azienda Ospedaliero Universitaria Parma |
| Stacy Gerle | Banner University Medical Centre |
| Emily Coxon | Baptist Health Louisville |
| Bruno Claro | Barts Hospital |
| Daniel Loverde | Billings Clinic |
| Namrata Patil | Brigham and Women’s Hospital |
| Vieri Parrini | Borgo San Lorenzo Hospital |
| Angela McBride | Brighton and Sussex Medical School |
| Kathryn Negaard | Brooke Army Medical Centre |
| Angela Ratsch | Bundaberg Hospital |
| Ahmad Abdelaziz | Cairo University Hospital |
| Juan David Uribe | Cardio VID |
| Adriano Peris | Careggi Hospital |
| Mark Sanders | Cedar Park Regional Medical Center |
| Dominic Emerson | Cedars-Sinai Medical Centre |
| Muhammad Kamal | Cengkareng Hospital |
| Pedro Povoa | Centro Hospitalar de Lisboa |
| Roland Francis | Charite-Univerrsitatsmedizi n Berlin |
| Ali Cherif | Charles Nicolle University Hospital |
| Sunimol Joseph | Children’s Health Ireland (CHI) at Crumlin |
| Matteo Di Nardo | Children’s Hospital Bambino Gesù |
| Micheal Heard | Children's Healthcare of Atlanta – Egleston Hospital |
| Kimberly Kyle | Children's Hospital – Los Angeles |
| Ray A Blackwell | Christiana Care Health System's Centre for Heart and Vascular Health |
| Michael Piagnerelli  Patrick Biston | CHU de Charleroi |
| Hye Won Jeong | Chungbuk National University Hospital |
| Reanna Smith | Cincinnati Children's |
| Yogi Prawira | Cipto Mangunkusumo Hospital |
| Giorgia Montrucchio | Città della Salute e della Scienza Hospital – Turin, Italy |
| Arturo Huerta Garcia | Clínica Sagrada Família |
| Nahikari Salterain | Clinica Universidad de Navarra |
| Bart Meyns | Collaborative Centre Department Cardiac Surgery, UZ Leuven |
| Marsha Moreno | Dignity Health Medical Group- Dominican |
| Rajat Walia | Dignity Health St. Joseph's Hospital and Medical Center (SJHMC) |
| Amit Mehta | Doernbecher Children’s Hospital |
| Annette Schweda | Donaustauf Hospital |
| Moh Supriatna | Kariadi Hospital Semarang |
| Cenk Kirakli | Suat Seren Chest Diseases and Surgery Practice and Training Centre |
| Melissa Williams | Duke University Hospital (Durham) |
| Kyung Hoon Kim | Eunpyeung St Mary's Hospital |
| Alexandra Assad | Fluminense Federal University |
| Estefania Giraldo | Fundación Clinica Shaio (Shaio Clinic) |
| Wojtek Karolak | Gdansk Medical University |
| Martin Balik | General University Hospital |
| Elizabeth Pocock | George Washington University Hospital |
| Evan Gajkowski | Giesinger Medical Centre |
| Kanamoto Masafumi | Gunma University Graduate School of Medicine |
| Nicholas Barrett | Guy's and St Thomas NHS Foundation Trust Hospital |
| Yoshihiro Takeyama | Hakodate City Hospital |
| Sunghoon Park | Hallym University Sacred Heart Hospital |
| Faizan Amin | Hamilton General Hospital |
| Fina Meilyana Andriyani | Hasan Sadikin Hospital (Paediatric) |
| Serhii Sudakevych | Heart Institute Ministry of Health of Ukraine |
| Angela Ratsch | Hervey Bay Hospital |
| Magdalena Vera | Hospital Clinico de la Pontificia Universidad Catolica |
| Rodrigo Cornejo | Hospital Clinico de la Universidad de Chile |
| Patrícia Schwarz  Ana Carolina Mardini | Hospital de Clínicas de Porto Alegre |
| Thais de Paula | Hospital Felicio Rocho |
| Ary Serpa Neto | Hospital Israelita Albert Einstein |
| Andrea Villoldo | Hospital Privado de Comunidad |
| Alexandre Siciliano Colafranceschi | Hospital Pro Cardíaco |
| Alejandro Ubeda Iglesias | Hospital Punta de Europa |
| Juan Granjean | Hospital Regional de Valdivia |
| Lívia Maria Garcia Melro  Giovana Fioravante Romualdo | Hospital Samaritano Paulista |
| Diego Gaia | Hospital Santa Catarina |
| Helmgton Souza | Hospital Santa Marta |
| Filomena Galas | Hospital Sirio Libanes |
| Rafael Máñez Mendiluce | Hospital Universitario de Bellvitge |
| Alejandra Sosa | Hospital Universitario Esperanza (Universidad Francisco Marroquin) |
| Ignacio Martinez | Hospital Universitario Lucus Augusti |
| Hiroshi Kurosawa | Hyogo Prefectural Kobe Children's Hospital |
| Juan Salgado | Indiana University Health |
| Beate Hugi-Mayr | Inselspital University Hospital |
| Eric Charbonneau | Institut Universitaire de Cardiologie et de Pneumologie de Quebec - Universite Laval |
| Vitor Salvatore Barzilai | Instituto de Cardiologia do Distrito Federal - ICDF |
| Veronica Monteiro | Instituto de Medicina Integral . Fernando Figueira (IMIP) |
| Rodrigo Ribeiro de Souza | Instituto Goiano de Diagnostico Cardiovascular (IGDC) |
| Michael Harper | INTEGRIS Baptist Medical Center |
| Hiroyuki Suzuki | Japan Red Cross Maebashi Hospital |
| Celina Adams | John C Lincoln Medical Centre |
| Jorge Brieva | John Hunter Hospital |
| George Nyale | Kenyatta National Hospital (KNH) |
| Faisal Saleem Eltatar | King Abdullah Medical City |
| Jihan Fatani | King Abdullah Medical City Specialist Hospital |
| Husam Baeissa | King Abdullah Medical Complex |
| Ayman AL Masri | King Salman Hospital NWAF |
| Ahmed Rabie | King Saud Medical City |
| Mok Yee Hui | KK Women's and Children's Hospital |
| Masahiro Yamane | KKR Medical Center |
| Hanna Jung | Kyung Pook National University Hospital |
| Ayorinde Mojisola Margaret | Lagos University Teaching Hospital |
| Newell Nacpil | Lung Center of the Philippines |
| Katja Ruck | Luxembourg Heart Center |
| Rhonda Bakken | M Health Fairview |
| Claire Jara | Maine Medical Centre (Portland Maine) |
| Tim Felton | Manchester University NHS Foundation Trust - Wythenshawe |
| Lorenzo Berra | Massachusetts General Hospital |
| Bobby Shah | Medanta Hospital |
| Arpan Chakraborty | Medica Super speciality Hospital |
| Monika Cardona | Medical University of South Carolina |
| Gerry Capatos | Mediclinic Parkview Hospital Dubai |
| Bindu Akkanti | Memorial Hermann - Texas Medical Centre |
| Abiodun Orija | Memorial Regional Hospital (Hollywood Florida) |
| Harsh Jain | Mercy Hospital of Buffalo |
| Asami Ito | Mie University Hospital |
| Brahim Housni | Mohammed VI University Hospital |
| Sennen Low | National Centre for Infectious Diseases |
| Koji Iihara | National Cerebral and Cardiovascular Center |
| Joselito Chavez | National Kidney and Transplant Institute |
| Kollengode Ramanathan | National University Hospital, Singapore |
| Gustavo Zabert | National University of Comahue |
| Krubin Naidoo | Nelson Mandela Children's Hospital |
| Ian Seppelt | Nepean Hospital |
| Marlice VanDyk  Sarah MacDonald | Netcare Unitas ECMO Centre |
| Shingo Ichiba | Nippon Medical School Hospital |
| Randy McGregor | Northwestern Medicine |
| Teka Siebenaler | Norton Children's Hospital |
| Hannah Flynn | Novant Health (NH) Presbyterian Medical Centre |
| Kristi Lofton | Ochsner LSA Health Shreveport |
| Toshiyuki Aokage | Okayama University Hospital |
| Kazuaki Shigemitsu | Osaka City General Hospital |
| Andrea Moscatelli | Ospedale Gaslini |
| Giuseppe Fiorentino | Ospedali dei Colli |
| Matthias Baumgaertel | Paracelsus Medical University Nuremberg |
| Serge Eddy Mba | Parirenyatwa General Hospital |
| Jana Assy | Pediatric and Neonatal Cardiac Intensive Care at the American University |
| Amelya Hutahaean | Pelni Hospital |
| Holly Roush | Penn State Heath S. Hershey Medical Centre |
| Kay A Sichting | Peyton Manning Children's Hospital |
| Francesco Alessandri | Policlinico Umberto, Sapienza University of Rome |
| Debra Burns | Presbyterian Hospital, New York/ Weill Cornell Medical Centre |
| Ahmed Rabie | Prince Mohammed bin Abdulaziz Hospital |
| Gavin Salt | Prince of Wales |
| Carl P. Garabedian | Providence Sacred Heart Children's Hospital |
| Jonathan Millar  Malcolm Sim | Queen Elizabeth II University Hospital |
| Adrian Mattke | Queensland Children’s Hospital |
| Danny McAuley | Queens University of Belfast |
| Jawad Tadili | Rabat University Hospital |
| Tim Frenzel | Radboud University Medical Centre |
| Yaron Bar-Lavie | Rambam Hospital |
| Aaron Blandino Ortiz | Ramón y Cajal University Hospital |
| Jackie Stone | Rapha Medical Centre |
| Alexis Tabah | Redcliffe Hospital |
| Antony Attokaran | Rockhampton Hospital |
| Michael Farquharson | Royal Adelaide Hospital |
| Brij Patel | Royal Brompton & Harefield NHS Foundation Trust |
| Derek Gunning | Royal Columbian Hospital |
| Kenneth Baillie | Royal Infirmary Edinburgh |
| Pia Watson | Sahlgrenska University Hospital |
| Kenji Tamai | Saiseikai Yokohamashi Tobu Hospital |
| Gede Ketut Sajinadiyasa  Dyah Kanyawati | Sanglah General Hospital |
| Marcello Salgado | Santa Casa de Misericordia de Juiz de Fora |
| Assad Sassine | Santa Casa de Misericórdia de Vitoria |
| Bhirowo Yudo | Sardjito Hospital |
| Scott McCaul | Scripps Memorial Hospital La Jolla |
| Bongjin Lee | Seoul National University Children's Hospital |
| Sang Min Lee | Seoul National University Hospital |
| Arnon Afek | Sheba Medical Center |
| Yoshiaki Iwashita | Shimane University Hospital |
| Bambang Pujo Semedi  Neurinda Permata Kusumastuti | Soetomo General Hospital (FK UNAIR) |
| Jack Metiva | Spectrum Health Western Governors University |
| Nicole Van Belle | St. Antonius Hospital |
| Ignacio Martin-Loeches | St James’s University Hospital |
| Lenny Ivatt | Swansea Hospital |
| Chia Yew Woon | Tan Tock Seng Hospital |
| Hyun Mi Kang | The Catholic University of Seoul St Mary Hospital |
| Timothy Smith | The Christ Hospital |
| Erskine James | The Medical Centre Navicent Health |
| Nawar Al-Rawas | Thomas Jefferson University Hospital |
| Yudai Iwasaki | Tohoku University |
| Kenny Chan King-Chung | Tuen Mun Hospital |
| Vadim Gudzenko | UCLA Medical Centre (Ronald Regan) |
| Beate Hugi-Mayr | Universitätsspital Bern, Universitätsklinik für Herz- und Gefässchirurgie |
| Fabio Taccone | Universite Libre de Bruxelles |
| Fajar Perdhana | University Airlangga Hospital (Adult) |
| Yoan Lamarche | University de Montreal (Montreal Heart Institute) |
| Joao Miguel Ribeiro | University Hospital CHLN |
| Nikola Bradic | University Hospital Dubrava |
| Klaartje Van den Bossche | University Hospital Leuven |
| Oude Lansink | University Medical Center Groningen |
| Gurmeet Singh | University of Aberta (Mazankowski Heart Institute) |
| Gerdy Debeuckelaere | University of Antwerp |
| Henry T. Stelfox | University of Calgary and Alberta Health Services |
| Cassia Yi | University of California at San Diego |
| Jennifer Elia | University of California, Irvine |
| Thomas Tribble | University of Kentucky Medical Center |
| Shyam Shankar | University of Missouri |
| Raj Padmanabhan | University of Pittsburgh Medical Centre |
| Bill Hallinan | University of Rochester Medical Centre (UR Medicine) |
| Luca Paoletti | University of South Carolina |
| Yolanda Leyva | University of Texas Medical Branch |
| Tatuma Fykuda | University of the Ryukyus |
| Jenelle Badulak | University of Washington in Seattle |
| Jillian Koch | University of Wisconsin & American Family Children's Hospital |
| Amy Hackman | UT Southwestern |
| Lisa Janowaik | UTHealth (University of Texas) |
| Deb Hernandez | Valley Children's Hospital (Madera) |
| Jennifer Osofsky | Vassar Brothers Medical Center (VBMC) |
| Katia Donadello | Verona Integrated University Hospital |
| Aizah Lawang | Wahidin Sudirohusodo Hospital |
| Josh Fine | WellSpan Health - York Hospital |
| Benjamin Davidson | Westmead Hospital |
| Andres Oswaldo Razo Vazquez | Yale New Haven Hospital |

**e-Appendix 4:** Case report form

Link to one version of the COVID-19 Critical Care Consortium Case Report Form Completion Guide: <https://static1.squarespace.com/static/5ec2086275fa7637bd6d9007/t/5f8521bec4442034cc3e9d6e/1602560463958/ISARIC+nCoV+COVID+19+CCC+Combined+Data+Completion+Guide+based+on+ISARIC+CRF+25.8.20.pdf>

**e-Appendix 5:** Case report definitions for HECTOR complications

The case report forms utilized in data collection defined HECTOR complications as:
**Thrombosis:**

- Ischemic stroke – may be a clinical diagnosis, with or without supportive radiological findings
- Myocardial ischemia – confirmed by an electrocardiogram (showing ischemic changes; e.g., ST depression or elevation) and/or cardiac enzye elevation
- Myocardial infarction – myocardial ischemia (MI) leading to injury/necrosis, diagnosed by clinical findings, altered electrocardiography, and elevated cardiac enzymes
- Deep venous thrombosis – Blood clots in deep veins of leg, pelvis or arm. Physician diagnosis based on clinical signs, and/or duplex ultrasonography, d-dimer blood test, contrast venography or magnetic resonance imaging (MRI).
- Pulmonary embolism – obstruction of pulmonary artery by thrombus, air, or fat. Physician diagnosis based on clinical signs, computed tomographic pulmonary angiography, and/or ventilation/perfusion scanning

**Hemorrhage:** defined by bleeding considered clinically significantly at a specific source; pulmonary, gastrointestinal, genitourinary, skin, and soft tissue, hemorrhagic stroke, pericardial effusion, or ECMO cannula site. If more than two sources were actively bleeding, the two most predominant sources were recorded by the site investigators. A standardized definition was not utilized but determined by treating clinician at each participating site.

**Disseminated intravascular coagulation (DIC; consumption coagulopathy; defibrination syndrome)**: defined by thrombocytopenia, prolonged prothrombin time, low fibrinogen, elevated D-dimer, and thrombotic microangiopathy
